# Secondary Metabolite Dereplication and Phylogenetic Analysis Identify Various Emerging Mycotoxins and Reveal the High Intra-Species Diversity in *Aspergillus flavus*

**DOI:** 10.3389/fmicb.2019.00667

**Published:** 2019-04-05

**Authors:** Valdet Uka, Geromy G. Moore, Natalia Arroyo-Manzanares, Dashnor Nebija, Sarah De Saeger, José Diana Di Mavungu

**Affiliations:** ^1^Center of Excellence in Mycotoxicology and Public Health, Faculty of Pharmaceutical Sciences, Ghent University, Ghent, Belgium; ^2^Department of Pharmacy, Faculty of Medicine, University of Prishtina, Prishtina, Kosovo†; ^3^Southern Regional Research Center, Agricultural Research Service, United States Department of Agriculture, New Orleans, LA, United States; ^4^Department of Analytical Chemistry, Faculty of Chemistry, Regional Campus of International Excellence “Campus Mare-Nostrum”, University of Murcia, Murcia, Spain

**Keywords:** *Aspergillus*, sexuality, chemical diversity, genetic diversity, mass spectrometry

## Abstract

*Aspergillus flavus* is one of the most important mycotoxigenic species from the genus *Aspergillus*, due to its ability to synthesize the potent hepatocarcinogen, aflatoxin B_1_. Moreover, this fungus is capable of producing several other toxic metabolites from the class of indole-tetramates, non-ribosomal peptides, and indole-diterpenoids. Populations of *A. flavus* are characterized by considerable diversity in terms of morphological, functional and genetic features. Although for many years *A. flavus* was considered an asexual fungus, researchers have shown evidence that at best these fungi can exhibit a predominantly asexual existence. We now know that *A. flavus* contains functional genes for mating, uncovering sexuality as potential contributor for its diversification. Based on our results, we reconfirm that *A. flavus* is a predominant producer of B-type aflatoxins. Moreover, this fungus can decisively produce AFM_1_ and AFM_2_. We did not observe any clear relationship between mating-type genes and particular class of metabolites, probably other parameters such as sexual/asexual ratio should be investigated. A dynamic secondary metabolism was found also in strains intended to be used as biocontrol agents. In addition we succeeded to provide mass spectrometry fragmentation spectra for the most important classes of *A. flavus* metabolites, which will serve as identification cards for future studies. Both, metabolic and phylogenetic analysis proved a high intra-species diversity for *A. flavus*. These findings contribute to our understanding about the diversity of *Aspergillus* section *Flavi* species, raising the necessity for polyphasic approaches (morphological, metabolic, genetic, etc.) when dealing with this type of complex group of species.

## Introduction

Among the casual agents of food and feed contamination, fungi from the genus *Aspergillus* play a crucial role. Within this genus, there are species that have the ability to produce a broad assortment of secondary metabolites, including mycotoxins. One very important group of fungi from *Aspergillus* section *Flavi* is known to produce the most potent carcinogenic compound of the mycotoxins, aflatoxin. In this connection, *Aspergillus flavus* is one of the most studied mycotoxigenic fungi (Bennett, 2010). Polyketide-derived mycotoxins like aflatoxin B_1_ have also been shown to possess additional hepatotoxic and immunosuppressive properties (Turner et al., 2003). Aflatoxin levels exceeding 0.025–15 μg/kg on food or feed commodities are strictly prohibited in the European Union (Commission regulation (EC) No 1881/2006). Moreover, *A. flavus* has the potential to act as a human pathogen, being the second most frequent cause, after *A. fumigatus*, of invasive and localized aspergillosis in immunocompromised patients (Hedayati et al., 2007).

Beyond aflatoxins, many other toxic secondary metabolites have been detected that contribute to the overall toxicity of *A. flavus*, including: other polyketides such as asparasones (Cary et al., 2014) and aflavarins (Cary et al., 2015a); non-ribosomal peptides such as piperazines (Forseth et al., 2013) and ditryptophenaline (Springer et al., 1977); hybrid molecules such as cyclopiazonic acids (Luk et al., 1977; Uka et al., 2017) and leporins (Arroyo-Manzanares et al., 2015; Cary et al., 2015b); and various indole-diterpenes such as aflatrem (Gallagher and Wilson, 1979; Nicholson et al., 2009) and aflavinines (Gallagher et al., 1980). Alpha-cyclopiazonic acid (α-CPA) (Uka et al., 2017) is another important *A. flavus* mycotoxin, which has been associated with different liver, kidney and gastrointestinal complications in animal health (Cole, 1986; Burdock and Flamm, 2000). Aflatoxins and α-CPA very often co-contaminate food and feed commodities (Cole, 1986; Lee and Hagler, 1991; Urano et al., 1992; Heperkan et al., 2012; Zorzete et al., 2013; Ezekiel et al., 2016). A more complete list of key fungal metabolites identified in *A. flavus*, based on the literature, is shown in Supplementary Figure S1 and Supplementary Table S1.

In general, *A. flavus* and related species within the ascomycete group of fungi are considered primarily asexual microorganisms (Debuchy and Turgeon, 2006). However, *A. flavus* exhibits high genetic diversity, which is reflected by its spatial distribution and by its large number of vegetative compatibility groups (VCGs), but also by diversity of its morphology (e.g., sclerotium size and number, conidium production) and physiology (e.g., secondary metabolite production) (Horn et al., 1996). Fungal individuals belonging to the same VCG are vegetatively compatible, so their hyphae have the ability to anastomose and possibly exchange genetic material via parasexual modes of recombination. Individuals from different VCGs are vegetatively incompatible, so hyphal fusion typically results in cell necrosis (Papa, 1973; Glass and Kaneko, 2003). Parasexuality occurs only among strains of the same VCG, which usually leads to isolation and homogeneity in metabolite patterns and morphology. This is the reason why individuals within a given VCG share similar morphological and physiological features (Grubisha and Cotty, 2009). The high level of diversity exhibited among *A. flavus* strains, even within localized populations, is difficult to explain by asexuality or parasexuality; therefore, sexuality and subsequent genetic recombination should be considered important for generating observed intra-specific diversity. Hence, research began in the 1990s aiming to investigate a possible sexual stage within the life cycles of filamentous ascomycetes (Metzenberg and Glass, 1990; Coppin et al., 1997). Moreover, it has been proposed that also horizontal gene transfer (HGT) has contributed significantly in the overall chemodiversity of *Aspergillus* species (Frisvad and Larsen, 2015). While parasexuality mainly occurs between strains of the same species, HGT is more likely to occur between phylogenetically distant species (Rank et al., 2011; Campbell et al., 2012). HGT of a secondary metabolite gene cluster can be a result of species occurring in the same habitat and sharing the same ecological challenges (Frisvad and Larsen, 2015). Specifically, through bioinformatics tools it was shown that *A. flavus* genome likely harbors more than 500 horizontally transferred genes, 41% of which were found to be engaged in physically linked gene clusters (Nguyen et al., 2015).

In 1998, evidence of cryptic sex was reported for an Australian *A. flavus* population, which suggested a potential risk for using non-aflatoxigenic strains of *A. flavus* as biopesticides (Geiser et al., 1998). Nearly a decade after, evidence of recombination was uncovered for a single *A. parasiticus* population (Carbone et al., 2007). Then it was determined that both *A. flavus* and *A. parasiticus* are heterothallic (self-infertile) fungi with individuals possessing either a *MAT1-1* or *MAT1-2* mating-type (MAT) gene (Ramirez-Prado et al., 2008), followed by additional evidence of recombination for a single population of *A. flavus* (Moore et al., 2009). *MAT1-1* encodes for a conserved alpha (α)-domain protein, whereas *MAT1-2* gene encodes for a conserved class of high mobility group (HMG)-domain protein (Turgeon and Yoder, 2000). Gene-disruption studies have shown that MAT genes are required for normal sexual development in heterothallic species (Debuchy and Turgeon, 2006). Moreover, is important to note that sexual reproduction is correlated with a significant increase expression of MAT genes and key genes of a pheromone-response MAP (mitogen-activated protein)-kinase signaling pathway involved in heterothallic outcrossing (Lengeler et al., 2000). Next, the sexual states for *A. parasiticus* (Horn et al., 2009a) and *A. flavus* (Horn et al., 2009b) were observed via experimental crosses. Finally, it was reported that the distribution of MAT genes in several global populations of *A. flavus* and *A. parasiticus* correlated with the amount of diversity within the population, as well as the distribution of chemotype profiles across a given population. As well, equal distributions (1:1 ratio) of MAT genes in populations of *A. flavus* and *A. parasiticus* were found to result in greater likelihood of uncovering evidence for the existence of sexual reproduction (Moore et al., 2013). These reports offered new insights into the biology of these agriculturally significant molds. In contrast to parasexuality, sexual reproduction happens between heterothallic individuals with opposite mating type (*MAT1-1* or *MAT1-2*) that belong to different VCGs, and may differ in their morphological and functional features (Horn et al., 2009a,b). Consequently, it was understood that asexual reproduction fixes metabolic profiles in fungal populations whereas sexual reproduction creates new VCGs with diversity of genetic composition and metabolite chemotypes (Moore et al., 2013). Even inter-specific hybridization via laboratory crosses has been observed, which offers more evidence of how these fungi may be capable of diversifying, morphologically and physiologically, as well as their overall adaptive ability (Olarte et al., 2015). Apart from laboratory experiments, *A. flavus* sexuality was also demonstrated in experimental field studies (Horn et al., 2013), and potential male/female roles were recently suggested for conidia/sclerotia as well as for each mating type (Horn et al., 2016).

The discovery of sexual states in aflatoxigenic fungi is expected to have a great impact in agriculture, ecology, and food safety. This is because mycotoxigenic species that undergo sexual recombination exhibit increased adaptability and may create more difficulties for their control and monitoring. Specifically, this may interfere with current strategies for developing biopesticides (pre-harvest biological controls) through use of non-aflatoxigenic *A. flavus* as a replacement for chemical pesticides (Abbas et al., 2011; Probst et al., 2011; Moore et al., 2013). This is highly relevant because its use as biological control is largely based on the paradigm that *A. flavus* is reproductively stable (i.e., asexual) (Ehrlich and Cotty, 2004). The long-term fate of *A. flavus* biocontrol strains in fields has not yet been fully addressed (Ehrlich, 2014), and the possibility of inheriting aflatoxin-producing ability for progeny that arise through sexual recombination is a possibility that should not be neglected after long-term treatment (Olarte et al., 2011).

Most investigations reporting correlations between sclerotium size, mating-type and VCGs with mycotoxin production have involved the aflatoxins because of their toxicity (Novas and Cabral, 2002; Amani et al., 2012; Moore et al., 2013). However, although *A. flavus* has potential to produce many other toxic secondary metabolites, most of the toxic effects of these secondary metabolites have been ascribed to aflatoxins. There is the possibility that some of these toxic metabolites are being produced in higher quantities by non-aflatoxigenic strains, which further might complicate the application of biocontrol strains in pre-harvest decontamination. Hence, the main objective of this study is to evaluate any relationship between secondary metabolite patterns in *A. flavus* and mating-type on one hand, and sclerotium size on the other hand, including not only aflatoxins but a much broader repertoire of secondary metabolites produced by this fungus. Thus, we aim to get a more complete picture of the mosaic of the *A. flavus* metabolome as a function of different genetic and morphological features. To address this issue, a secondary metabolite screening program was performed on different biological strains of *A. flavus* deploying an identification methodology based on high-resolution mass spectrometry (HRMS). Indeed, screening of biological samples through full-scan HRMS can result in an unlimited number of putative metabolites that can be identified. However, the final confirmation of these metabolites is still seen as the major bottleneck in MS-based analytical approaches. MS/MS or fragmentation data allows a stronger absolute metabolite identification. In order to obtain these types of data (both accurate mass measurements and MS/MS) within a single analytical run, an UHPLC hybrid qTOF (quadrupole Time-of-Flight) mass spectrometer was used. In addition, complementary identification tools such as isotope similarity and elution order were applied. Where possible, the retention (RT) time and MS/MS spectra of putative compounds were matched with authentic reference standards, although standards are not available for all of the known or suspected metabolites.

## Materials and Methods

### Chemicals and Materials

Methanol (MeOH), LC–MS grade, was obtained from Biosolve (Valkenswaard, Netherlands), and HPLC-grade MeOH was obtained from VWR International (Zaventem, Belgium). Ethyl acetate (EtOAc), dichloromethane (DCM) and acetone [dimethyl ketone (DMK)] were purchased from Acros Organics (Geel, Belgium). Sigma-Aldrich (Bornem, Belgium) supplied ammonium formate (HCOONH_4_). Formic acid (HCOOH, Merck, Darmstadt, Germany) was used. Ultrapure H_2_O, used during these analyses was produced in-house with a Milli-Q Gradient System (Millipore, Brussels, Belgium). Ultrafree^®^-MC centrifugal filter units (0.22 μm) from Millipore (Bedford, MA, United States) were used for filtration and reconstitution of fungal extracts. Sigma-Aldrich was our source for fungal growth media, such as agar, corn steep solids, dextrose, peptone, sucrose, yeast extract, dipotassium hydrogen phosphate trihydrate (K_2_HPO_4_⋅3H_2_O), magnesium sulfate heptahydrate (MgSO_4_⋅7H_2_O), and iron(II) sulfate heptahydrate (FeSO_4_⋅7H_2_O). Other compounds necessary for this study included Triton X-100, potassium chloride (KCl), and sodium nitrate (NaNO_3_), which were obtained from Merck. Standards of AFB_1_, AFB_2_, AFG_1_, AFG_2_, AFM_1_, and ST (sterigmatocystin) were purchased from Oskar Tropitzsch (Marktredwitz, Germany).

### Strains and Sample Preparation

The *A. flavus* strains used in this study are listed in Table 1. Conidia from each strain were inoculated onto 10 cm petri plates containing solid Wickerham medium (≈25 ml per plate), which is comprised of the following: 2.0 g yeast extract, 3.0 g peptone, 5.0 g corn steep solids, 2.0 g dextrose, 30 g sucrose, 2 g NaNO_*3*_, 1 g K_2_HPO_4_⋅3H_*2*_O, 0.5 g MgSO_*4*_⋅7H_*2*_O, 0.2 g KCl, 0.1 g FeSO_4_⋅7H_*2*_O, 15 g agar per liter (pH 5.5). All cultures were incubated at 28°C in the dark for 7 days. The resulting fungal colonies were cut into small pieces with a scalpel and subsequently transferred to 500 ml screwcap Ehrlenmeyer flasks. Metabolites were extracted with 30 ml MeOH:DCM:EtOAc (10:20:30, v/v/v). The samples were agitated for 60 min on an Agitelec overhead shaker (J. Toulemonde & Cie, Paris, France). A total of 4 ml of extract was transferred to a glass tube and evaporated under a stream of nitrogen gas. The residue was reconstituted with 200 μl MeOH, and centrifuged in an Ultrafree^®^-MC centrifugal device for 5 min at 14,000 × *g*.

**Table 1:** List of *A. flavus* strains used in this study.

SRRC ID	Other designations	MAT	SM^a^	SGb^b^	Source
38	NRRL A-12268; ATCC 26938	1	U	SB	Turkey feed, Washington D.C.
141	ATCC 24109; Pep-70-1hle	2	U	SB	Black pepper, Louisiana
144	NRRL A-16464; SU25	1	U	SB	Cottonseed, Louisiana
150	TR 955	2	U	S/L	Cottonseed, Arizona
151	TR UNK3	2	U	SB	Cottonseed, Arizona
167	NRRL 3357; CBS128202; ATCC 200026	1	L	L	Peanut cotyledon, Arizona
283	NRRL 5918; SRRC 296	1	U	SB	Corn, Minnesota
295	NRRL 3537; SRRC 284; ATCC 9643	2	U	SB	Shoe sole, Papua New Guinea
1000F	GH flavus #257	2	U	L	Cottonseed, Arizona
1006	012981-7	2	U	SB	Cottonseed, Arizona
1020	061181-10	1	U	SB	Cottonseed, Florence
1021	061281-5	2	S	SB	Cottonseed, Florence
1055	–	1	U	L	Cottonseed, Arizona
1071	–	1	U	L	Cottonseed, Arizona
1098	–	1	U	L	Cottonseed, Arizona
1118	–	1	U	SBG	Cottonseed, Arizona
1187	–	2	L	L	Cottonseed, Arizona
1299	P. Cotty #12	2	S	SB	Soil, Arizona
1356	–	1	U	L	Dried bacon, Croatia
1357	–	1	U	SB	Dried bacon, Croatia
1533^c^	AF36; NRRL 18543	2	L	SB	Cottonseed, Arizona
1534^c^	Afla-Guard^®^; NRRL 21882	2	L	U	Peanut, Georgia
1540	BS07	2	L	SB	Bayside, Texas
1541	CA 1	2	S	SB	Pistachio, California
1543	CA 3	2	S	SB	Pistachio, California
1544	CA 4	2	S	SB	Pistachio, California
1545	CA 5	1	L	SB	Pistachio, California
1547	CA 7	1	L	SB	Pistachio, California
1552	CA 12	2	U	SB	Pistachio, California
1553	CA 13	2	S	SB	Pistachio, California
1554	CA 14	2	L	L	Pistachio, California
1557	CA 17	1	S	SB	Pistachio, California
1558	CA 18	1	L	SB	Pistachio, California
1559	CA 19	2	L	L	Pistachio, California
1565	CA 26	2	L	L	Pistachio, California
1566	CA 28	2	S	SB	Pistachio, California
1568	CA 32	1	S	SB	Pistachio, California
1571	CA 37	1	S	SB	Pistachio, California
1573	CA 39	1	S/L	SB	Pistachio, California
1574	CA 40	1	L	SB	Pistachio, California
1575	CA 41	1	L	SB	Pistachio, California
1576	CA 42	2	S	SB	Pistachio, California
1578	CA 44	2	S	SB	Pistachio, California
1591	SF-1	2	S	SBG	Rain forest soil, Nigeria
1626	SF-32	2	U	SB	Cowpea, Nigeria
1637	SF-48	2	U	SB	Bread, Nigeria
2000	–	1	U	L	Cottonseed, Arizona
2001	–	1	U	L	Cottonseed, Arizona
2033	FER 2749	1	U	SBG	Peanut, Australia
2035	FRR 2748	1	U	L	Peanut, Australia
2111	–	1	U	SB	
2114	ATCC 15546; FRR 3339	2	U	L	Moldy wheat, Illinois
2115	VDR 15	2	U	L	Sunflower seed, South Africa
2118	N-63-9	2	U	L	Dried fish, Indonesia
2524	FC017; T-19	1	L	L	Dead termites, China


*MAT, mating type; SM, sclerotium morphotype; SG, sclerotium genotype; ^*a*^L = large sclerotia (>400 μm); S = small sclerotia (<400 μm); U = unknown or non-sclerotial; ^*b*^Genotype based on size of deletions within the *aflF/aflU* region of the aflatoxin gene cluster: L = 1000 bp deletion; S = 1500 bp deletion; SBG = no deletion; U = unknown or not discernable; ^*c*^Commercially available biocontrol agent; SRRC, Southern Regional Research Center, New Orleans, LA, United States; NRRL, Northern Region Research Laboratory, Peoria, IL, United States; ATCC, American Type Culture Collection, Manassas, VA, United States; FRR, Food Research Laboratory, CSIRO, North Ryde, NSW, Australia; CBS, Centraalbureau voor Schimmelcultures, Utrecht, Netherlands.*

### HRMS Analysis

The experiments were carried out using a hybrid qTOF MS instrument, the AB SCIEX TripleTOF^®^4600 (Concord, Ontario, Canada), equipped with DuoSpray^TM^ and coupled to an Eksigentekspert^TM^ ultraLC 100-XL system. The DuoSpray^TM^ ion source [consisting of both electrospray ionization (ESI) and atmospheric pressure chemical ionization (APCI) probes] was operated in both, positive and negative ESI modes (ESI^+^/ESI^-^). The APCI probe was used for automated mass calibration using the calibrant delivery system (CDS). The CDS injects a calibration solution matching the polarity of ionization, and calibrates the mass axis of the TripleTOF^®^ system in all of the scan functions used (MS and/or MS/MS). The qTOF HRMS method consisted of a full scan TOF survey (dwell time 100 ms, 100–1600 Da) and a maximum number of eight information-dependent acquisition (IDA) MS/MS scans (dwell time 50 ms). The MS parameters were as follows: curtain gas (CUR) 25 psi, nebulizer gas (GS 1) 50 psi, heated gas (GS 2) 60 psi, ion spray voltage (ISVF) 5.5 kV, interface heater temperature (TEM) 500°C, collision energy (CE) 10 V and declustering potential (DP) 70 V. For the IDA MS/MS experiments, a CE of 35 V was applied with a collision energy spread (CES) of 15 V. An Eksigentekspert^TM^ ultraLC 100-XL system was used for separation. The column was a ZORBAX RRHD Eclipse Plus C18 (1.8 μm, 2.1 mm × 100 mm) from Agilent Technologies (Diegem, Belgium). The mobile phase consisted of H_2_O:MeOH (95:5, v/v) containing 0.1% HCOOH and 10 mM HCOONH_4_ (solvent A) and MeOH:H_2_O (95:5, v/v) containing 0.1% HCOOH and 10 mM HCOONH_4_ (solvent B). The gradient elution program for UHPLC-qTOF HRMS analyses was applied as follows: 0–0.5 min: 0% B, 0.5–7 min: 0–99% B, 7–9 min: 99% B, 9–10 min: 99–0% B, 10–14 min: 0% B. The flow rate was 0.4 ml/min. The column temperature was set at 40°C and temperature of the autosampler was 4°C. A volume of 5 μl of each sample was injected. The instrument was controlled by Analyst^®^ TF 1.6 software, while data processing was carried out using PeakView^®^ 2.0 and MasterView^TM^ 1.0 software (all from AB Sciex).

### Metabolite Screening Workflow

Accurate mass qTOF HRMS data were used to identify *A. flavus* metabolites, putatively by dereplication, using a customized database. Afterward, accurate mass HRMS data were combined with a careful evaluation of fragmentation spectra, to ascertain the presence of previously identified *A. flavus* metabolites and to establish an unambiguous identification strategy for subsequent screening work. The employed analytical methodology involved an untargeted data acquisition (consisting of full scan TOF HRMS survey and IDA MS/MS scans) and the processing of data using both targeted and untargeted approaches. Where possible, reference standards were used, and in this way compounds were identified in the fungal extracts by comparison of retention time (RT), accurate mass HRMS and HRMS/MS data with the authentic samples. A careful investigation and interpretation of fragmentation data was the basis for the identification of metabolites as described further.

### Molecular Studies of 55 Examined *A. flavus* Strains

DNA was first extracted from each of the 55 *A. flavus* strains by inoculating their respective spore suspensions into each of 75 ml potato dextrose broth (PDB) in 250 ml Erlenmeyer flasks, which were placed on an orbital shaker (30°C, 135 rpm) for 24 h. Once substantial spore germination occurred, the resulting mycelia were vacuum filtered through a Büchner funnel using Miracloth to retain the mycelial tissue, and a wash was performed with sterile water. Mycelial tissue (200 mg) was collected into a 1.5 mL microcentrifuge tube, and a DNA extraction protocol was followed using the MasterPure Yeast Purification kit (Epicentre Biotechnologies, Madison, WI, United States). Optical density readings were performed on extracted genomic DNA using a Nano Drop (ND-1000) spectrophotometer, and dilutions (if necessary) were made in preparation for PCR amplification of unlinked genomic loci: acetamidase (*amdS*), beta-tubulin (*benA*), calmodulin (*cmdA*), and tryptophan synthase (*trpC*). The use of sequence variation at multiple, unlinked, conserved genomic loci allows for clone correction and is known as multi-locus sequence typing (MLST), and has been used in studies where haplotype associations account for genetic recombination (Olarte et al., 2011, Olarte et al., 2013; Moore et al., 2013). Another genomic region of interest spanned the area between the *aflF* and *aflU* genes from the aflatoxin gene cluster, which in *A. flavus* reportedly contains deletions associated with loss of G-aflatoxin production as well as with sclerotium morphotype (Ehrlich et al., 2004). For this study, no other aflatoxin cluster genes were examined. The mating-type (MAT) locus was also examined for these strains. Amplification of the *aflF/aflU* region and the MAT locus were solely diagnostic, and were not used for MLST in this study. The primers for each locus are listed in Supplementary Table S2. We used GoTaq Colorless Master Mix (Promega) to perform the PCR amplifications. Variable cycle conditions for each primer set are also listed in Supplementary Table S2. To test the success of PCR amplification, electrophoresis was performed using 5 μl of PCR product on a 1.5% agarose gel in TAE buffer. DNA amplicons were purified using a QIAquick PCR purification kit (Qiagen, Hilden, Germany) before sequencing. Nucleotide sequences for each locus were aligned, cleaned and trimmed using Sequencher version 5.4.2 (Gene Codes Corporation, Ann Arbor, MI, United States). The alignments for *amdS* (290 bp), *benA* (486 bp), *cmdA* (628 bp), and *trpC* (235 bp) sequences were then exported as nexus files for concatenation, haplotype associations through MLST, and phylogenetic analysis using SNAP Workbench (Price and Carbone, 2005). Once the nexus files were imported into SNAP Workbench, they were converted into phylip files using SNAP Map and then concatenated using SNAP Combine (Aylor et al., 2006). The concatenated alignment (1639 bp sequences) was collapsed into haplotypes using SNAP Map with the options of recoding indels (insertions/deletions) and exclusion of sites that violated the infinite sites model. Phylogenetic inference was based on heuristic parsimony searches in PAUP^∗^ 4.0 (Swofford, 1998), which involved a branch-swapping algorithm. An *A. parasiticus* strain, SU1, was used as the outgroup taxon for our phylogenetic inference. Resulting most parsimonious trees were viewed with branch lengths based on mutational differences. Bootstrap analysis was conducted using PAUP^∗^ 4 in SNAP Workbench, using default parmeters of 500 replicate iterations. Bootstrap values lower than 70 were considered unsupportive and would not be shown on the inferred tree.

## Results

### Metabolite Identification

#### Polyketides

Since aflatoxins remain one of the most studied groups of mycotoxins, with clearly established analytical parameters, their identification was straightforward. The identification of AFB_1_, AFB_2_, AFG_1_, and AFG_2_ is described in Supplementary Figure S2. Interestingly, we were able to demonstrate the presence of aflatoxin M_1_ (AFM_1_) and aflatoxin M_2_ (AFM_2_) as native metabolites in some of our fungal cultures of *A. flavus* (see Supplementary Figure S3 for details), although these compounds are mainly reported as metabolic derivatives of B-type aflatoxins found in different animal and human biological fluids (Prandini et al., 2009). A perfect match between tandem MS spectra of AFM_1_ and AFM_2_ found in our data (Supplementary Figure S3) and previous reports was observed (Plattner et al., 1984; Cavaliere et al., 2006; Huang et al., 2010; Díaz et al., 2011). The identified aflatoxins were confirmed by comparison of theirs MS data and RTs with those of authentic standards, highlighting the suitability of our proposed dereplication strategy for analyte detection and identification. In addition, we detected an aflatoxin derivative (*[m+H]/z* 331.0820, Δ = 2.4 ppm) having the same chemical composition as AFM_2_, but eluting just after it. The MS fragmentation pattern of this metabolite pointed to aflatoxin B_2a_ or another functional isomer of AFM_2_. Its MS/MS spectrum clearly shows the loss of the hydroxyl group as a water molecule generating a very prominent ion at m/z 313 (Supplementary Figure S3C). Subsequently, the fragment ion at m/z 313 follows an identical fragmentation pathway as described for AFB_1_. This fragmentation pattern is perfectly in agreement with previous reports (Inoue et al., 2013; Rushing and Selim, 2017). However, without an authentic standard we could not assign it decisively as aflatoxin B_2a_.

**FIGURE 1:**
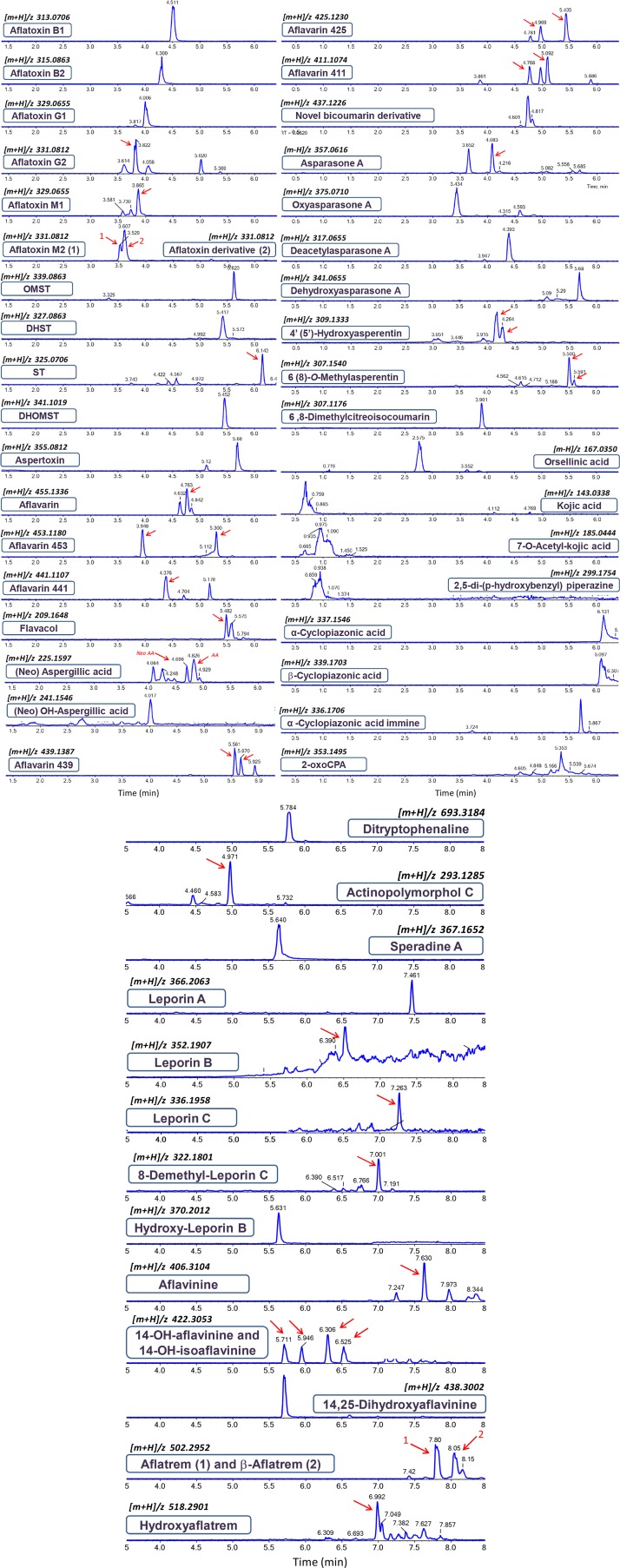
Extracted ion chromatograms (EICs) and elution order of the *Aspergillus flavus* metabolites detected in this study. Red arrows indicate the correct retention time.

Four other ultimate and penultimate precursors of aflatoxins could also be detected in fungal cultures of different strains, including: sterigmatocystin (ST), dihydrosterigmatocystin (DHST), *O-methylsterigmatocystin* (OMST), and dihydro-*O*-methylsterigmatocystin (DHOMST). All of the aforementioned precursors belong to the xanthone chemical subgroup of aflatoxin intermediates, possessing almost an identical core structure with very few minor chemical differences. These xanthone intermediates in the aflatoxin biosynthetic pathway share comparable and logical fragmentation patterns (Supplementary Figure S4). The fragmentation pattern we observed for these compounds is in strong accordance with previous publications (Bloom et al., 2007; Pfeiffer et al., 2014; Buiarelli et al., 2015). In this line of investigation, another derivative of OMST was putatively identified as being aspertoxin (*[m+H]/z* 355.0819, Δ = 1.8 ppm), which is actually a bisfuran oxygenated analog of OMST displaying a 16 Da difference between homologous fragment ions, such as 340 (324), 327 (311), 322 (306), 311 (295), and 294 (278) (Supplementary Figure S4E). Other aflatoxin precursors from the xanthone group of metabolites, such as demethylsterigmatocystin (DMST) and dihydro-demethylsterigmatocystin (DHDMST), could not be detected in our observations. Early stage intermediates in aflatoxin biosynthesis such as anthraquinones and other polyketide progenitors were not subject of this study. The extracted ion chromatograms (EICs) of the different *A. flavus* metabolites detected in this study are shown in Figure 1.

Another important group of *A. flavus* polyketides are bicoumarins, belonging to the C3-C8′-linked chemical class of fungal bicoumarins recognized for their anti-insectan properties. Moreover, these molecules possess a very intriguing pathway of biosynthesis involving a dimerization mechanism of two coumaryl subunits via oxidative phenol-coupling reactions. These reactions are fundamentally controlled by dirigent P450 proteins that enable regio- and stereo-selective C-C-cross coupling of coumaryl monomers (Hüttel and Müller, 2007; Gil Girol et al., 2012). Six bicoumaryl analogs and four monomers are reportedly produced via the aflavarin gene cluster (cluster #39) in *A. flavus* (Cary et al., 2015a) (Supplementary Figure S1 and Supplementary Table S1). Those six bicoumarins (i.e., aflavarin and derived compounds) could also be detected in the present study (Supplementary Figures S5, S6); the MS data were as previously reported (Cary et al., 2015a) thereby unambiguously confirming the identity of these compounds.

A group of anthraquinones, different from those produced in the aflatoxin biosynthetic cascade, is reportedly synthesized via a polyketide gene cluster (cluster #27) in *A. flavus* (Cary et al., 2014). We detected and confirmed the identity of four of these compounds, i.e., asparasone A (*[m-H]/z* 357.0617, Δ = 3.5 ppm), oxyasparasone A (*[m+H]/z* 375.0723, Δ = 3.5 ppm), dehydroxyasparasone A (*[m+H]/z* 341.0669, Δ = 3.8 ppm) and deacetylasparasone A (*[m-H]/z* 315.0511, Δ = 2.4 ppm) (Supplementary Figure S7). This identification was also supported by findings from previous reports (Malysheva et al., 2014).

As described in Supplementary Figure S8, a series of isocoumarin metabolites were also identified in our investigations, including four derivatives of asperentin (6-*O*-methylasperentin, 8-*O*-methylasperentin, 4′-hydro- xyasperentin and 5′-hydroxyasperentin) and 6,8-dimethylcitreoisocoumarin. Asperentin and its derivatives chemically are structural analogs of isocoumarin and possess a 6′-methyltetrahydropyran-2′-methylene-2′-yl moiety attached to the C8 position of the isocoumaryl core system. Each of the pairs 6-*O*-methylasperentin/8-*O*-methylasperentin and 4′-hydroxyasperentin/5′-hydroxyasperentin represents functional isomers eluting at very close RT points (Figure 1) and possessing almost identical MS/MS spectra (Supplementary Figure S8).

Kojic acid is a small yet well-known metabolite produced by various *Aspergillus* species with wide application in cosmetics. The presence of this molecule (whose diagnostic fragment ions have been previously reported by Varga et al. (2013) and that of its acetylated derivative was confirmed in some of the examined *A. flavus* cultures as described in Supplementary Figure S9. Moreover, in ESI negative mode we were able to detect orsellinic acid in several *A. flavus* isolates (*[m-H]/z* 167.0354, Δ = 2.4 ppm). The MS/MS spectrum shown in Supplementary Figure S9C was in full accordance with spectra from a previously reported study of *A. nidulans* (Sanchez et al., 2010). To the best of our knowledge this is the first report of orsellinic acid in *A. flavus*.

#### Non-ribosomal Peptides

Within the heterogeneous class of non-ribosomal peptide metabolites produced by *A. flavus*, the aspergillic acid group comprises a number of closely-related pyrazinones with pronounced antibiotic activity (Dutcher, 1947, 1958). Moreover, this group of metabolites is recognized as hydroxamate siderophores of *A. flavus* due to the iron-chelating activity exerted by the cyclic hydroxamic acid motif in their chemical scaffolds (Assante et al., 1981; Zhu et al., 2011). The presence of these comppunds (i.e., aspergillic acid, neoaspergillic acid, hydroxyaspergillic acid, and neohydroxyaspergillic acid) in the analyzed *A. flavus* cultures was demonstrated as described in Supplementary Figure S10. The obtained spectra are in accordance with data from literature (Assante et al., 1981; Perry et al., 1984). RT assignment for isomeric compounds was supported by literature, where it has been shown that *neo*-forms of this class of pyrazinones elute earlier compared to their corresponding analogs. None of the metal-chelating complexes characteristic for this class of compounds, such as ferriaspergillin or aluminiumaspergillin, could be detected in our observations. Two homologous NRPS-like gene clusters in *A. flavus* (*lna* and *lnb*) have been shown to be responsible for the convergent biosynthesis of a pair of diastereomeric piperazines known as the *cis*- and *trans*-2,5-di-(*p*-hydroxybenzyl) piperazines. These compounds were detected in the investigated strains as described in Supplementary Figure S11A. We also deteted a compound (see Supplementary Figure S11B) that was previously demonstrated to be linked to the same biosynthetic machinery as the above piperazines (Forseth et al., 2013).

Ditryptophenaline, a homodimeric diketopiperazine alkaloid, has been identified as an important secondary metabolite in several *A. flavus* strains (Springer et al., 1977). This class of metabolites contains two hexahydropyrroloindole substructures, connected at vicinal quaternary stereocenters (C3-C3′ linkage), to form the dimeric molecules. Due to their astonishing chemical architecture, this and other related dimeric natural products exhibit various biological activities. In this context, it has been shown that ditryptophenaline inhibits substance P receptors, implying a potential analgesic or anti-inflammatory application. Moreover, it was recently demonstrated that a single cytochrome P450, *DtpC*, located within an *A. flavus* NRPS gene cluster, is responsible not only for pyrroloindole ring formation but also for catalyzing the C3-C3′ dimerization of N-methylphenylalanyl-tryptophanyl diketopiperazine monomers into a homodimeric final product (Saruwatari et al., 2014). The identification of ditryptophenaline in our study was quite straightforward due to its unique mode of fragmentation. The observed fragmentation pattern is described in Supplementary Figure S11C and is in accordance with previous studies (Springer et al., 1977; Barrow and Sedlock, 1994).

#### Hybrid Metabolites (PK-NRPs)

Chemical entities sculpted by mixed PKS/NRPS enzymatic systems have always been characterized as variable in terms of structural scaffolds and bioactive features. Genome sequencing has demonstrated that *A. flavus* contains two hybrid PKS/NRPS gene clusters (clusters #55 and #23). Cluster #55 is responsible for the synthesis of α-CPA (Seshime et al., 2009), while cluster #23 has been shown to be responsible for the production of leporins, a group of 2-pyridone metabolites (Cary et al., 2015b). The CPA class of alkaloids represents a heterogeneous group of indole-tetramate mycotoxins with pronounced implications in food safety and was indeed investigated in the present study. The identification of five of these CPA-type alkaloids (i.e., α-CPA, β-CPA, α-CPA imine, 2-oxoCPA, and speradine A) is described in Supplementary Figure S12. Screening of other CPA-type mycotoxins in different *A. flavus* strains, as well as their fragmentation behaviors, has been previously discussed in detail (Uka et al., 2017). With regard to the biosynthetic products of cluster 23, leporin B (a cyclic hydroxamic acid) was shown to be especially important due to its pronounced iron-chelating activity. MS analysis (Supplementary Figure S13) confirmed the production of leporin A (*[m+H]/z* 366.2078, Δ = 3,8 ppm), leporin B (*[m+H]/z*352.1905, Δ < 0,1 ppm), leporin C (*[m+H]/z* 336.1968, Δ = 2,9 ppm), 8-demethyl-leporin C (*[m+H]/z* 322.1814, Δ = 4,0 ppm), iron-trioxoleporin B (*[m+H]/z* 1107.4674, Δ = -1.5 ppm) as well as a hydroxylated derivative of leporin B (*[m+H]/z* 370.2012, Δ < 0,1 ppm) by the different *A. flavus* cultures investigated. The MS data were in accordance with previous reports (Arroyo-Manzanares et al., 2015; Cary et al., 2015b).

#### Indole-Diterpenoids

For many years it has been known that *A. flavus* produces metabolites with potent tremorgenic activity. Through subsequent investigations, it was determined that this class of molecules share a basic indole-diterpene ring system generated by the fusion of a polycyclic diterpene, derived from a geranylgeranyldiphosphate (*ggpp*), and an indole moiety with tryptophan origin. These intriguing molecules are synthesized by terpene/terpenoid cyclases, very complex groups of enzymes which contribute crucially to the tremendous structural diversity of this large family of natural products. In the *A. flavus* secondary metabolome, two different chemical groups of indole-diterpenes have been described, aflavinines and aflatrem, along with their derivatives. Aflavinines possess a very interesting and sterically-congested chemical structure, in which the indole moiety and the diterpenoid ring system are coupled via a single C-C chemical bond. We identified aflavinine (*[m+H]/z* 406.3107, Δ = 0,74 ppm), 14-hydroxyaflavinine (*[m+H]/z* 422.3053, Δ < 0,1 ppm) and its isomers, as well as 14,25-dihydroxyaflavinine (*[m+H]/z* 438.3017, Δ = 3.2 ppm) in cultures of the *A. flavus* strains analyzed in this study. A detailed description of the confirmation of the identity of those compounds is given in Supplementary Figure S14. Regarding 14-hydroxyaflavinine, interestingly, there are four metabolites eluting at different RTs (RT 5.7, 5.9, 6.3, and 6.5 min), all having the same exact mass and sharing the fragmentation pattern of monohydroxyaflavinine. This is most likely due to the position of the double bond in ring C, as well as the epimerization of the hydroxyl group at C14, resulting in four isomers of monohydroxyaflavinine: 14-hydroxyaflavinine, 14-*epi*-hydroxyaflavinine, 14-hydroxyisoaflavinine and 14-*epi*-hydroxyisoaflavinine. The elution order of these metabolites cannot be determined unambiguously; however, reports from the literature indicate that iso-forms elute earlier, as assigned in Figure 1. A similar mode of fragmentation has been encountered for 14,25-dihydroxyaflavinine with three consecutive losses of hydroxyl functionalities, followed by the chronological fragmentation of the C_20_-terpenoid moiety (Supplementary Figure S14C).

Aflatrem is another *A. flavus* metabolite possessing a prenylated heptacyclic indole-diterpenoid skeleton with very potent tremorgenic toxicity. Moreover, it was demonstrated that aflatrem is synthesized through a very complex and convergent biosynthetic pathway, governed by two different gene clusters located on separate chromosomes of *A. flavus* (Nicholson et al., 2009). In our investigation of the *A. flavus* strains, a compound with *[m+H]/z* 502.2952, cooresponding to aflatrem (Δ < 0.1 ppm) could be conspicuously detected in significant abundance. Accurate mass data, combined with the fragmentation behavior, decisively confirmed the presence of aflatrem as described in Supplementary Figure S15. Apart from aflatrem, β-aflatrem is another structural isomer described in the literature, which was also detected in our study. The only structural difference between these two isomers is the position of the dimethylallyl moiety in the indole core system, thus exhibiting the same fragmentation pattern as described for aflatrem. Based on previous reports, aflatrem elutes earlier in reverse-phase chromatography (Nicholson et al., 2009). Similarly, hydroxyaflatrem (*[m+H]/z* 518.2917, Δ = 3.0 ppm), an oxygenated analog of aflatrem, could be detected in several strains of *A. flavus*. Assignment of the position of this hydroxyl group in the aflatrem prototype structure is not so straightforward, due to the number of possible options. Nevertheless, MS/MS data (Supplementary Figure S15B) indicated that this functionality most likely is attached in the indole moiety of the core structure. The identity of other aflatrem derivatives such as paspaline, paspalinine, or paspalicine could not be decisively confirmed in our experiments.

### Distribution of Metabolites as a Function of Genetic and Morphological Features

**FIGURE 2:**
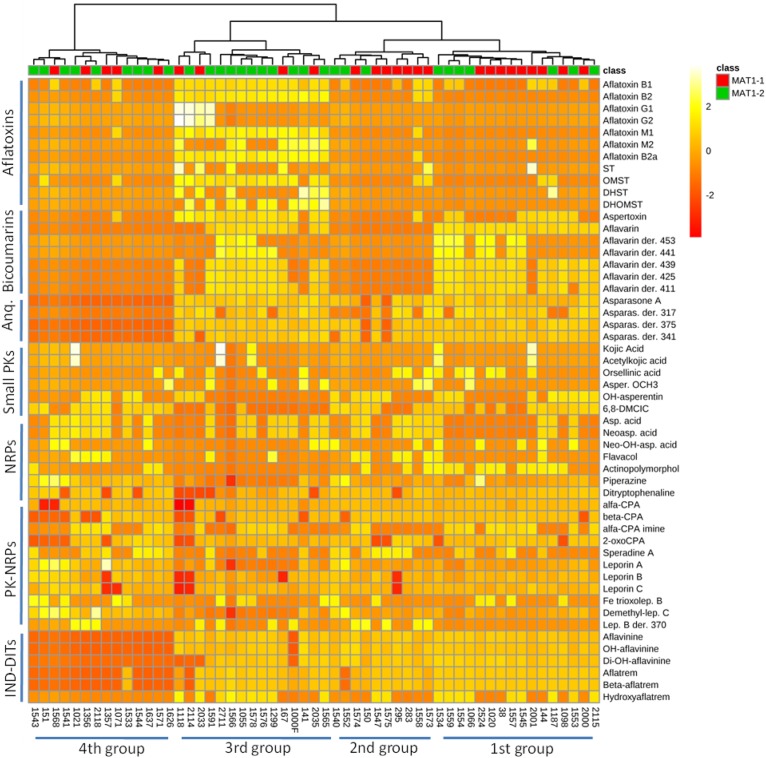
Heat map representing metabolite distribution in 55 *A. flavus* isolates. On the top of the figure, a dendogram is depicted, clustering *A. flavus* isolates as function of mating-type genes (*MAT1-1* and *MAT1-2*) and metabolite occurrence. This figure was generated using the online metabolomics platform *MetaboAnalyst 3.0* after the data were treated accordingly using sum normalization, log transformations and Pareto scaling. The data table was prepared using area under the curve (AUC) for each chromatographic peak of respective metabolites. Anq, anthraquinones; PKs, polyketides; NRPs, non-ribosomal peptides; IND-DITs, indole-diterpenoids; ST, sterigmatocystin; OMST, *O-methylsterigmatocystin*; DHST, dihydro-ST; DHOMST, dihydro-OMST; Asparas., asparasone A; Asper. OCH_3,_ asperentin methyl-ether; 6,8-DMCIC, 6,8-dimethylcitreoisocoumarin; Asp. acid, aspergillic acid; Neoasp. acid, neoaspergillic acid; α-CPA, alfa-cyclopiazonic acid; Lep. B, leporin B.

**FIGURE 3:**
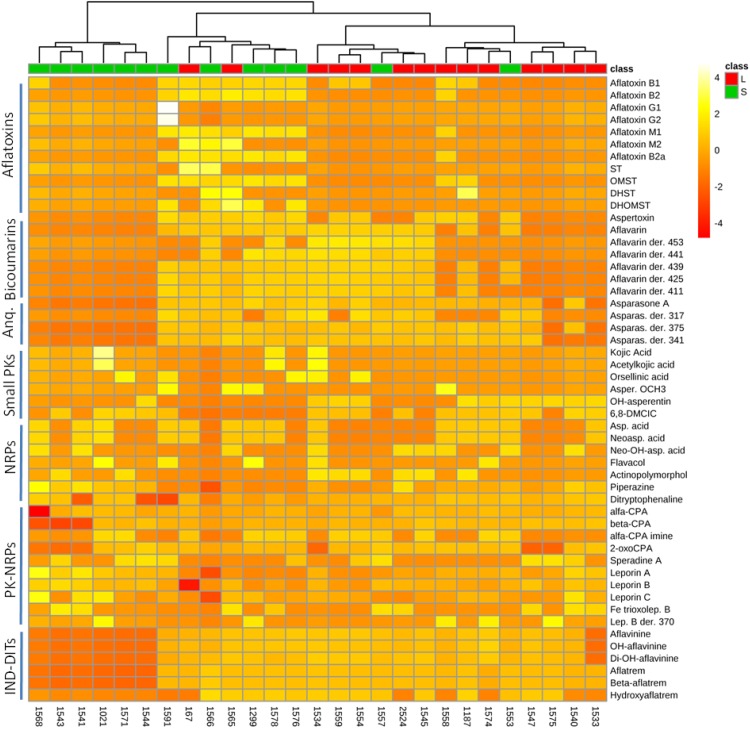
Heat map representing metabolite distribution in 27 *A. flavus* isolates with assigned sclerotium morphotype. On the top of the figure, a dendogram is depicted, clustering *A. flavus* isolates as function of sclerotium morphotype: L (large sclerotia > 400 μm) and S (small sclerotia < 400 μm). This figure was generated using the online metabolomics platform *MetaboAnalyst 3.0* after the data were treated accordingly using sum normalization, log transformations and Pareto scaling. The data table was prepared using AUC foreach chromatographic peak of respective metabolites. Anq, anthraquinones; PKs, polyketides; NRPs, non-ribosomal peptides; IND-DITs, indole-diterpenoids; ST, sterigmatocystin; OMST, *O-methylsterigmatocystin*; DHST, dihydro-ST; DHOMST, dihydro-OMST; Asparas., asparasone A; Asper. OCH_3,_ asperentin methyl-ether; 6,8-DMCIC, 6,8-dimethylcitreoisocoumarin; Asp. acid, aspergillic acid; Neoasp. acid, neoaspergillic acid; alfa-CPA, alfa-cyclopiazonic acid; Lep. B, leporin B.

Occurrence of *A. flavus* secondary metabolites as function of mating-type genes in different strains is determined as shown in Figure 2 and Supplementary Tables S3–S5. The heat map data visualization on a targeted list of metabolites shows the clustering of the 55 *A. flavus* isolates into four groups (clades). From right to left of the heat map, the first group seems to produce bicoumarins, anthraquinones, NRPs, PK-NRPs, and indole-diterpenoids but less often aflatoxins. The second group, seems to lack the ability in producing bicoumarins in addition to aflatoxin absence. The third group produces almost all classes of secondary metabolites. Whereas in the 4th group a higher frequency for NRPs and PK-NRPs is observed but less polyketides and indole-diterpenoids. Based on this limited list of known metabolites, there was no clear correlation/clustering between mating-type genes and secondary metabolite production. On the other hand distribution of metabolites as function of sclerotium size showed a better clustering pattern (Figure 3).

### Molecular Studies of 55 Examined *A. flavus* Strains

Some of the examined *Aspergillus* strains had been previously accessioned for one or more of the four conserved loci of interest. A list of all GenBank accession numbers is shown in Supplementary Table S6. Of the 55 *A. flavus* strains examined, four strains failed to properly amplify and/or sequence for one of the four loci. Despite repeated attempts, strain 150 failed to result in a useable sequence for the *trpC* locus, while strains 1568, 1574, and 1575 failed to result in useable sequences for the *cmdA* locus. The sequences obtained for one strain, 1118, across all four loci appeared highly polymorphic in relation to those of the other strains. BLAST query of each locus for 1118 revealed it to be an *A. nomius* strain. This can only be explained by an inaccurate morphological identification, so 1118’s species identification has been changed in the SRRC’s fungal database to accurately reflect our genomic identifications. Results of BLAST queries for each strain’s locus sequences are shown in Supplementary Table S7. Most of the strains were found to be *A. flavus*; however, nucleotide sequences for some strains appeared to have variable species designations across the four loci (1576, 1591, 2000, and 2033). Amplification of their *aflF/aflU* genomic regions revealed deletions that show 1576 to be an *A. flavus* S-strain (1500 bp deletion) and 2000 to be an *A. flavus* L-strain (1000 bp deletion). This indicates that strains 1576 and 2000 should not produce G aflatoxins, yet they share species identity for the *benA* locus with *A. minisclerotigenes* and *A. parvisclerotigenus*, respectively, which reportedly produce B and G aflatoxins (Frisvad et al., 2005; Pildain et al., 2008). It is possible for *A. parvsclerotigenus* to lack production of G aflatoxins (Frisvad et al., 2005). Strains 1591 and 2033 do produce G aflatoxins, therefore their respective species accessions for the *benA* locus (*A. parvisclerotigenus*) and *amdS* locus (*A. minisclerotigenes*) could be accurate. The amplification of intact *aflF/aflU* regions (i.e., no deletions present) in 1591 and 2033 further corroborated their production of G aflatoxins.

**FIGURE 4:**
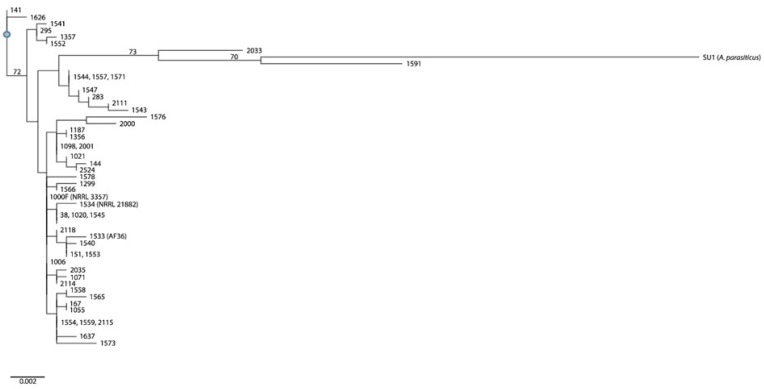
Phylogenetic tree inferred for the *A. flavus* isolates examined in this study. Strain SU1 is the outgroup taxon, *A. parasiticus*. Phylogenetic inference is based on the concantenation of four unlinked genomic loci (amdS, benA, cmdA, and trpC).

One of the consequences of a strain not being represented for one or more loci in a concatenated sequence is exclusion from population analyses, therefore, the four strains mentioned above (150, 1568, 1574, 1575) were not included in our multi-locus inferences. We also left out the newly-identified *A. nomius* strain, 1118. The haplotype designations from our revised sample of 51 *Aspergillus* strains (including SU1) can be seen in Table 2. A total of 43 haplotypes (i.e., individuals) resulted from collapsing 51 concatenated sequences. Some haplotypes include multiple isolates that share sequence homology across all four unlinked genomic loci. A potential bottleneck with population analysis results when high complexity of sequence data proves too much for certain analysis software. We experienced this during phylogenetic analysis using the stand-alone version of SNAP Workbench. Bootstrap analysis would not complete. To addres this issue we used an online version of the software that allows runs to be placed on a cluster of networks, which accelerates analysis. We were able to complete bootstrap analysis (based on 1000 runs), and then view/export our tree using RAxML 8 (Stamatakis, 2014), accessible through the CIPRES RESTful API (Miller et al., 2015), implemented in the Mobyle SNAP Workbench v1.55 (Monacell and Carbone, 2014); the tree was visualized using an online portal software known as T-BAS v. 2.1 (Carbone et al., 2017). Figure 4 shows inferred phylogenetic associations examined in this study. No clear cladal distinctions could be made on the basis of mating type, sclerotium morphotype, *aflF/aflU*-deletion type, or metabolic profile. Clade I includes the *A. parasiticus* Type strain, SU1. It was inferred to share a most recent common ancestor with strain 1591, which is a B+G aflatoxing-producing S-strain that had been identified as both *A. flavus*, based on BLAST query of its *amdS*, *cmdA* and *trpC* loci, and as *A. parvisclerotigenus* based on its *benA* locus. Bootstrap support was moderate, having a value of 70. Strain 1591 was the only one in this entire study found to produce leporin A, a compound that has only been described in *A. flavus* and *A. leporis* (Frisvad et al., 2019). Strain 2033 shares a common ancestor with that of SU1 and 1591, having a boostrap value of 73. Although there is much metabolic overlap across the three strains, including the production of M aflatoxin, 2033 fails to produce any of the examined bicoumarins or non-ribosomal peptides. Strain 2033 is a B+G aflatoxin producing S-strain that had been identified as both *A. flavus*, based on BLAST query of its *benA*, *cmdA* and *trpC* loci, and as *A. minisclerotigenes* based on its *cmdA* locus. The remaining strains in Clade I are mostly non-aflatoxigenic and share the S-type deletion in their *aflF/aflU* region, supporting their lack of G-aflatoxin production, but not all exhibit the S-morphotype for sclerotium production. Strain 1547 produces L-type sclerotia, while strains 283 and 2111 failed to produce any sclerotia. Strain 2111 is a high producer of B and M_1_ aflatoxins. Three of the strains in this clade (1544, 1557, 1571) share sequence identity across all four conserved loci, are all of the same sclerotium morphotype, have the same S-type deletion in *aflF/aflU*, and are all non-aflatoxigenic. One observed difference among them relates to mating type, since strain 1544 is *MAT1-2* while the others are *MAT1-1*. Other observed differences are metabolic, such as our observation of 1544 producing only two isocoumarin compounds and six PK-NRP hybrid molecules, or that only strain 1557 produced any of the examined bicoumarin and indole-diterpene compounds, or that only 1571 produced orsellinic acid.

**Table 2:** Haplotype identities for 51 *Aspergillus* strains examined in this study.

Haplotype^a^	Strain(s)
1	SU1 (*A. parasiticus*)
2	2033
3	1071
4	1554, 1559, 2115
5	1591
6	1565
7	1573
8	1543
9	2111
10	1578
11	283
12	1055
13	167
14	1533 (AF36)
15	151, 1553
16	1540
17	144
18	1534 (NRRL 21882)
19	1020, 1545, 38
20	1299
21	1357
22	1187
23	1356
24	1626
25	1637
26	1576
27	2000
28	1552
29	1566
30	1541
31	1006
32	2118
33	295
34	141
35	1544, 1557, 1571
36	1547
37	1558
38	1021
39	1098, 2001
40	2035
41	1000F (NRRL 3357)
42	2114
43	2524


*^*a*^Haplotypes based on four concatenated genomic loci: *amdS*, *benA*, *cmdA*, and *trpC.**

Clade II encompasses most of the examined isolates (*n* = 35), representing 29 haplotypes, and includes the two commercially-available biocontrol strains (AF36 = 1533 and NRRL 21882 = 1534). Although this clade has a nearly equal distribution of *aflF/aflU*-deletion types (800 bp/L-type = 18; 1500 bp/S-type = 17), no partitioning was observed based on this phenotype. Similarly, no branch partitioning could be observed that related to mating type, sclerotium morphotype or aflatoxin chemotype. Interestingly, one strain (2114) has the L-type deletion in *aflF/aflU*, but was observed to produce G aflatoxins. This strain produced no detectable bicoumarin compounds, nor did it produce aflatrems, NRPs or PK-NRPs. Clades I and II share a most recent common ancestor, but there was a lack of bootstrap support for this. There was, however, bootstrap support for the convergence of Clades I and II with Clade III, having a value of 72.

Clade III includes only four strains (295, 1357, 1541, 1552). These strains mainly share one phenotype, which is the S-type deletion in their *aflF/aflU* regions. With regard to aflatoxin producing ability, 1357 and 1541 are non-aflatoxigenic, while 295 and 1552 produce a small amount of B_1_ aflatoxin. In comparing the two non-aflatoxigenice strains, 1357 lacks production of all but two of the examined polyketides (Supplementary Table S3), and 1541 produces only one polyketide (orsellinic acid). Strain 1541 produces greater numbers and quantities of aspergillic acid compounds (Supplementary Table S4), but no flavocol or ditryptophenaline (all non-ribosomal peptides). Nor does it produce hybrid molecules beyond α-CPA, leporins B and C, and iron trioxoleporin B. Neither strain produces indole-diterpenes (Supplementary Table S5). Aflatoxin B1 producers, 295 and 1552, share similar polyketide production profiles, except for the production of asparsone 317 and orsellinic acid (only in 295) and 6,8-dimethylcitreo isocourmarin (only in 1552). Strain 1552 only produces the non-ribosomal peptide ditryptophenaline, and it lacks production of β-CPA among the hybrid molecules, while 295 lacks production of leporins B and C (Supplementary Table S4). Among the indole-diterpenes (Supplementary Table S5), both strains produce aflavinines, but only 295 produces aflatrems. There is also no clustering based on mating type, as only 1357 is a *MAT1-2* strain while the other three are *MAT1-1*.

Clade IV includes two strains (141, 1626) that appear basal to the remaining sample population. These strains share the same mating type, *aflF/aflU* deletion type (S), and both fail to produce sclerotia. Among the polyketides, strain 141 was observed to produce B and M aflatoxins, while 1626 is non-aflatoxigenic (Supplementary Table S3). Neither strain produces bicoumarin compounds, and only 141 produces the asparasone compounds and orsellinic acid. Both strains produce the isocoumarin compound known as asperentin-6(8)-methyl ether, but only 1626 produces 6,8-dimethylcitreo isocourmarin. Both strains produce only a single non-ribosomal peptide (ditryptophenaline), but 1626 produces a greater number of hybrid molecule compounds (Supplementary Table S4). Strain 1626 produces no indole-diterpenes, while 141 produces all but hydroxyaflatrem (Supplementary Table S5).

## Discussion

Occurrence of various metabolites in different *A. flavus* individuals was unambiguously determined using a dereplication approach, based on HRMS data combined with tandem MS spectra thereby allowing a confident identification of several compounds. Dereplication is an especially important and useful tool for natural product chemists to avoid re-isolation of already known metabolites, thus saving time and resources. Dereplication analytical methodologies are most often implemented through hybrid HRMS mass spectrometers, which are automated approaches known to be fast, robust and have a strong identification potential (Wolfender et al., 2000; Fredenhagen et al., 2005; Nielsen et al., 2011; El-Elimat et al., 2013). Less often, other analytical techniques such as UV spectroscopy and NMR have been used in dereplication strategies (Bobzin et al., 2000; Wolfender et al., 2003). In the present study we constructed our in-house database with specific *A. flavus* secondary metabolites, gathering from literature all possible parameters such as chemical formulas, MS/MS spectra and elution order, which then were integrated in MS-software packages to proceed with the screening work. As expected, we observed a scattered and heterogeneous occurrence of compounds in different strains of *A. flavus*, which correlates with its high genetic diversity. This was especially true for the polyketide class of metabolites (Supplementary Table S3). Publications in the literature indicate great variability between different strains of *A. flavus* in their ability to produce aflatoxins (Hesseltine et al., 1968; Geiser et al., 2000; Ehrlich et al., 2007). In general, aflatoxigenic *A. flavus* isolates are predominantly known as B-type aflatoxin producers that are incapable of producing G-type aflatoxins (Taber and Schroeder, 1967; Ehrlich et al., 2007). In this study of 55 *A. flavus* strains, 28 were aflatoxin producers. Only four of those 28 isolates produced all (B-, G-, and M-types) of the aflatoxins; 13 isolates produced only B- and M-type aflatoxins; in one strain we detected production of only AFB_1_ and AFB_2a_; 10 strains produced B-type aflatoxins alone; and 27 strains produced no detectable aflatoxins. We did not observe a single strain that could produce G- or M-type aflatoxins without also producing B-type aflatoxins. Our detection of aflatoxins M_1_ and M_2_ in fungal cultures is of particular interest because M-type aflatoxins were originally found, and are most often reported being sampled, in milk and other biological fluids (Holzapfel et al., 1966; Masri et al., 1967; Hesseltine et al., 1968; Stubblefield et al., 1970; Diener et al., 1987). These findings indicate that occurrence of M-type aflatoxins in different food matrices does not necessarily require animal or human involvement for their production, because we observed these hydroxylated aflatoxins as endogenous metabolites of *in situ A. flavus*. The ability of *Aspergillus* spp. to produce M-aflatoxins has already been previously demonstrated (Yabe et al., 2012). The authors induced AFM_1_ and AFM_2_ production in *A. parasiticus* by feeding experiments. Feeding of *A. parasiticus* with aspertoxin (12c-hydroxyOMST) resulted in the production among others of these two metabolites by the fungus. Furthermore, cell-free experiments using the microsomal fraction of *A. parasiticus* and aspertoxin also led to the production of AFM_1_ and AFM_2_, indicating that aspertoxin is a precursor of these metabolites. Interestingly, incubation of either the microsomal fraction or OrdA enzyme-expressing yeast with OMST or DHOMST resulted in the production of aspertoxin together with the M-type aflatoxins, indicating that the OrdA enzyme catalyzes both 12c-hydroxylation reaction from OMST/DHOMST to aspertoxin/dihydroaspertoxin and the subsequent reaction from aspertoxin/ dihydroaspertoxin to AFM_1_/AFM_2_. Production M-aflatoxins by the investigated *A. flavus* strains should theorically be possible through mediation of OrdA enzyme; our results decisively confirming this, highlighting the power and the usefulness of mass spectrometry untargeted analysis in providing a global and better insight into the different metabolites that could be present in a sample. Several survey studies have reported presence of AFM_1_ in different food matrices (Chala et al., 2014; Sulyok et al., 2015; Abdallah et al., 2017). Furthermore, based on our findings it seems that *A. flavus* more frequently produces M-type aflatoxins compared to aflatoxin G_1_ and G_2_. Actually, the ability/inability of *A. flavus* isolates to produce G-type aflatoxins may relate to a deletion in the encoding gene for a cytochrome P450 enzyme (*aflU)* within the pathway of the aflatoxin biosynthetic cluster (Ehrlich et al., 2004). More precisely, these deletions have been reported in the *aflF/aflU* region of the aflatoxin gene cluster. Three out of the four G-producing strains examined in this study had no deletion in their *aflF/aflU* regions, which justifies their G-producing ability. Our finding of G-aflatoxin production by strain 2114, despite possessing a 1000 bp deletion in its *aflF/aflU* region, should be explored further. Perhaps there is another gene present that allows for the production of G aflatoxins when the *aflU* gene is deleted.

Aflatoxin B- and G-producing isolates from Thailand, which morphologically resemble *A. flavus*, were definitively assigned as a novel clade of *A. nomius* (Ehrlich et al., 2007). Perhaps this morphological similarity is why strain 1118 was misidentified as *A. flavus*. It is becoming more commonly accepted that *A. flavus* S-type isolates, that are also B+G aflatoxin producers, belong to one of the three phylogenetically related species: *A. minisclerotigenes* (Pildain et al., 2008), *A. parvisclerotigenus* (Frisvad et al., 2005, 2019), and *A. korhogoensis* (Carvajal-Campos et al., 2017). Several recent studies have characterized “novel” species that produce B and G aflatoxins such as *A. mottae*, *A. transmontanensis*, *A. sergii*, *A. pseudonomius*, and *A. novoparasiticus* (Varga et al., 2011; Gonçalves et al., 2012; Soares et al., 2012). Moore and co-workers are seeking to determine whether or not these novel species, which exhibit striking morphological and metabolic similarities to *A. flavus* and *A. parasiticus*, are actually hybrid species through genomic sequencing of the Type strains for all aflatoxigenic species (Moore et al., 2015a,b, 2016). We may find that aflatoxigenic fungi from Section *Flavi* form a species complex whose constituent “species” are capable of inter-specific recombination, and that recombination between closely-related *Aspergillus* species contributes to the genus’ genetic and metabolic diversity. As well, evolutionary relationships for their respective metabolomes can be further investigated.

Regarding other polyketide metabolites, we found a consistent presence of bicoumarins and anthraquinones. Among non-ribosomal peptides, ditryptophenaline was the most prevalent metabolite, occurring in 47 of our examined strains, followed by the aspergillic acid group of mycotoxins (Supplementary Table S4). Regarding hybrid polyketide-aminoacid molecules, both the CPA and 2-pyridone groups of metabolites were highly present (Supplementary Table S4). Detailed occurrence of CPA-type alkaloids in this set of *A. flavus* isolates was reported previously (Uka et al., 2017). Leporin B, a cyclic hydroxamic acid iron chelator, was the most frequent metabolite observed, from within the 2-pyridone class of metabolites. However, we determined the presence of indole-diterpenoids in 42 of the examined strains (Supplementary Table S5). Among these 42, 38 individuals were capable of producing both the aflavinine- and aflatrem-type of indole-diterpenoids.

All *A. flavus* soil populations have the potential to include strains representing two sclerotial morphotypes: the L-strain isolates with average sclerotial size greater than 400 μm, and the S-strain isolates with sclerotial size less than 400 μm (Cotty, 1997). S-strain isolates reportedly produce higher levels of aflatoxins, more abundant sclerotia and fewer conidia (Probst et al., 2012). In this study we were unable to definitely ascertain sclerotium production for all examined strains; nevertheless, based on the limited number of strains the dendogram clustering analysis show a quite satisfactory grouping of individual isolates by function of sclerotium morphotype (Figure 3). This indicates that sclerotium morphotype is likely an important morphological feature influencing production of secondary metabolites. The *aflF/aflU* region of the aflatoxin cluster has been associated with sclerotium morphotype (Ehrlich et al., 2004), but we found at least one contradiction to this association in our examination of these *A. flavus* strains.

Although mycotoxin contamination may occur during transport or storage of food products, pre-harvest contamination from fungal infections in plant hosts is another source of mycotoxins. In order to reduce aflatoxin contamination during pre-harvest periods, a number of mitigation strategies like fungicide application, development of resistant plants and biological control have been explored. Biocontrol approaches, involving the introduction of non-aflatoxigenic *A. flavus* isolates into agricultural fields to displace aflatoxin-producing strains, have been tested extensively in the last two decades (Dorner et al., 2003; Abbas et al., 2011; Atehnkeng et al., 2014). Apart from aflatoxins, the inability of *A. flavus* biocontrol agents to produce other relevant mycotoxins, among which are α-CPA, ditryptophenaline, aflatrem and aflavinines, is highly desirable due to the unknown long-term and cumulative toxicological effects of these metabolites. Two commercially available biocontrol agents, AF36 (SRRC strain 1533) and the component strain in Afla-Guard^®^, NRRL 21882 (SRRC strain 1534), were included in this study. AF36 was originally isolated from a cotton field in Arizona and is approved for application on various commodities in Arizona and California. Loss of aflatoxigenicity in AF36 is the result of a nonsense mutation in its *aflC*, a pathway gene involved in aflatoxin biosynthesis (Ehrlich and Cotty, 2004). AF36 has an otherwise complete aflatoxin cluster. NRRL 21882 lacks the entire aflatoxin gene cluster (Chang et al., 2005). Apart from CPA-type mycotoxins, most of which were recently investigated (Uka et al., 2017), we could detect many more metabolites as part of the secondary metabolome of these aforementioned biological controls. Secondary metabolites like ditryptophenaline, aflatrem-type tremorgens, the aspergillic acid group of mycotoxins and 2-pyridones were detected in both biocontrol agents. However, aflavarin and aflavinine-type tremorgenic compounds were exclusively detected in 1534 (Supplementary Tables S3–S5). Our findings suggest that these biocontrol agents are very active in producing secondary metabolites with different toxicological profiles, an issue which should not be ignored when considering their application to agricultural fields.

As was stated above, populations of *A. flavus* are characterized by considerable diversity in terms of morphological, functional, and genetic features, which is clearly supported by the large number of VCGs and different mycotoxin chemotypes. The large number of VCGs for *A. flavus* should significantly limit the frequencies of hyphal anastomosis, except when the necessity to undergo sexual out-crossing (i.e., to circumvent unfavorable conditions) relaxes those incompatibility barriers and contributes greatly to high genetic diversity among individual isolates. It has been reported that species populations such as *A. tamarii* or *A. parasiticus* possess lower VCG diversity, and consequently experience a higher frequency of hyphal contact (Horn and Greene, 1995). The high variation between *A. flavus* strains is not easily justified in the absence of sexuality. Although for many years *A. flavus* was considered an asexual fungus, researchers have shown evidence that at best these fungi can exhibit a predominantly asexual existence. We now know that *A. flavus* contains functional genes for mating. The expression of these genes has been demonstrated at the mRNA and protein levels. Following the same logic, it would be of great interest to decipher the expression of these genes in relation to secondary metabolism, aiming to establish any type of relationship between a single or a group of secondary metabolites with one or the other MAT gene. In addition, elucidation of these correlations between MAT genes and secondary metabolism is of great importance since the general indications that sexual reproduction is occurring between strains with differing capacities for mycotoxin production. Based on our analyses, inferred from a limited list of known metabolites, we could not observe any particular relationship between any of the mating-type genes and secondary metabolite production (Figure 2). This could be also due to the fact that other possible unknown metabolites not included in our list are responsible for making the real difference. Or maybe these results are just reflecting the reality that individual mating-type genes do not influence expression of any specific secondary metabolite gene cluster. In this context, it could be that comparing the secondary metabolomes of individual strains possessing opposite mating-type genes is not the right strategy to judge about the real contribution of sexuality in secondary metabolite chemical diversity. Hence, other biological parameters in *A. flavus* populations like sexual/asexual ratio could be a more relevant feature to track the correlations between sex and chemical diversity. An example could be to compare the secondary metabolome of a field population of *A. flavus* isolates with an almost equal distribution of *MAT1-1* and *MAT1-2* genes (sexual recombination) with another population which predominantly undergoes asexual recombination (unequal occurrence of *MAT1-1* and *MAT1-2* genes). In this way, we would be able to evaluate more precisely the real impact of sexuality in secondary metabolite and mycotoxin production.

Phylogenetic analysis did not offer much resolution for the examined strains. Lack of strong boostrap support and partitioning of haplotypes based on a particular phenotype, underscore the complexity of *A. flavus* strains with regard to morphology, genetics and production of metabolites. Many of the strains examined are from different geographic locations and/or different hosts. Perhaps the best inferences are made from sample populations that are considered panmictic (i.e., from the same location and host type), which might add more power to the analyses. Our findings support the importance of incorporating a holistic approach to strain/species identification. If we had not undertaken molecular research, we would have assumed all of our strains were *A. flavus*. What fungal researchers have to be careful of is assuming that just because two strains produce different metabolites it does not mean they are different species. The same should be said for assuming that two strains that are similar in appearance are the same species. With the new found concepts of intra- and inter-specific hybridization for *Aspergillus* fungi, the possibilities are endless. Therefore, species identifications should encompass morphological, metabolic and genetic comparisons. Furthermore, genetic associations should include multiple loci (both individual and combined).

## Author Contributions

JM, GM, and VU, conceived and designed the experiments. JM and GM, generated the data. VU, GM, NA-M, and JM, analyzed the data. VU, GM, DN, SS, and JM, wrote the manuscript.

## Conflict of Interest Statement

The authors declare that the research was conducted in the absence of any commercial or financial relationships that could be construed as a potential conflict of interest.
