# Supplementary material for: Secondary Metabolite Dereplication and Phylogenetic Analysis Identify Various Emerging Mycotoxins and Reveal the High Intra-Species Diversity in Aspergillus flavus
Source: Front Microbiol. 2019 Apr 5;10:667. doi: 10.3389/fmicb.2019.00667 (PMC6461017; doi:10.3389/fmicb.2019.00667)
Supplement: Supplementary file 1 [file Data_Sheet_1.docx]

Figure S1. Chemical structures of different *A. flavus* metabolites described in literature: A) Polyketides (PKs); B) Non-ribosomal peptides (NRPs); C) Hybrid molecules (PK-NRPs); D) Indole-diterpenoids. DMST (demethylsterigmatocystin); DHDMST (dihydro-demethylsterigmatocystin); DHST (dihydrosterigmatocystin); OMST (*O*-methylsterigmatocystin); DHOMST (dihydro-*O*-methylsterigmatocystin); α-CPA (α-Cyclopiazonic acid).

Figure S1. *Continued.*

Figure S1. *Continued.*

Figure S2. High resolution MS/MS spectra of: A) Aflatoxin B_1_; B) Aflatoxin B_2_; C) Aflatoxin G_1_; D) Aflatoxin G_2_. The MS/MS spectra were acquired in IDA (information dependent acquisition) mode using a CE (collision energy) of 35 V with a collision energy spread (CES) of 15 V.

The presented MS/MS data were used for the identifications of peaks eluting at RT 4.5, 4.3, 4.0 and 3.8 min corresponding to measured exact mass at *[m+H]^+^/z* 313.0717 (C17H13O6, Δ=3.1 ppm), *[m+H]^+^/z* 315.0870 (C17H15O6, Δ=1.6 ppm), *[m+H]^+^/z* 329.0672 (C17H13O7, Δ=3.5 ppm), and m/z 331.0803 (C16H15O7, Δ=−2.1 ppm), respectively. The peaks were identified as AFB_1_, AFB_2_, AFG_1_ and AFG_2_, respectively. Typical and diagnostic fragment ions of AFB_1_ (m/z 298, 285, 270, 257, 241), AFB_2_ (m/z 287, 272, 259, 243), AFG_1_ (m/z 311, 283, 255, 243) and AFG_2_ (m/z 313, 303, 275, 285, 245) were observed; overall, the MS/MS spectra and RTs were in agreement with those of aflatoxin standards.

Figure S3. High resolution MS/MS spectra and their putative structural fragments of: A) Aflatoxin M_1_;

B) Aflatoxin M_2_; C) Aflatoxin derivative 331. The MS/MS spectra were acquired in IDA (information dependent acquisition) mode using a CE (collision energy) of 35 V with a collisionenergy spread (CES) of 15 V.

AFM_1_ and AFM_2_ are hydroxylated analogues of AFB_1_ and AFB_2_, respectively. More precisely, a hydroxyl functionality is attached to the bisfuran moiety of each of the B-type aflatoxins. In this regard, a 16 Da difference can be noticed when comparing key structural fragments between B-type and M-type aflatoxins. Therefore, AFM_1_ (*[m+H]^+^/z* 329.0655, Δ<0.1 ppm) exhibited an MS/MS spectrum with characteristic fragment ions at m/z 311, 301, 283, 273, 259, 241 and 229, whilst AFM_2_ (*[m+H]^+^/z* 331.0821, Δ=2.7 ppm) generated a tandem MS spectrum with respective fragments at 313, 303, 285, 273, 259, 241 and 229.

Figure S4. High resolution MS/MS spectra: A) Sterigmatocystin (ST); B) Dihydrosterigmatocystin (DHST); C) *O*-Methylsterigmatocystin (OMST); D) Dihydro-*O*-methylsterigmatocystin (DHOMST); E) Aspertoxin. The MS/MS spectra were acquired in IDA (information dependent acquisition) mode using a CE (collision energy) of 35 V with a collision energy spread (CES) of 15 V.

Based on their structural similarities, these xanthone intermediates in the aflatoxin biosynthetic pathway must share comparable and logical fragmentation patterns. The MS/MS spectrum of the protonated ion of ST (*[m+H]^+^/z* 325.0706, Δ<0.1 ppm) exhibits its most characteristic fragment ions at m/z 310, 297, 281 and 253 (Spectrum A). The compound DHST (*[m+H]^+^/z* 327.0877, Δ=3.6 ppm) has a 2 Da difference from ST due to the fact that the double bond in the bisfuran moiety of DHST is saturated. This mass difference of 2 Da is reflected in the MS/MS spectrum of DHST through homologous fragment ions at m/z 312, 299, 283 and 255 (Spectrum B). The same analogy and logic of fragmentation applies in the case of *O*-methyl derivatives of ST (OMST) and DHST (DHOMST). OMST (*[m+H]^+^/z* 339.0875, Δ=2.9 ppm) represents an *O*-methyl analogue of ST, thus exhibiting a 14 Da difference with regard to the parent ion as well as to other characteristic fragments. The MS/MS spectrum of OMST shows prominent fragment ions at m/z 324, 311, 306, 295 and 267 (Spectrum C). On the other hand, DHOMST (*[m+H]^+^/z* 341.1029, Δ=2.6 ppm) has a 2 Da difference from OMST and a 14 Da difference compared to DHST, as depicted in its MS/MS spectrum with typical fragment ions at m/z 326, 313, 308, 297 and 269 (Spectrum D).

Figure S5. High resolution MS/MS spectra: A) Aflavarin; B) Aflavarin-439; C) Aflavarin-425; D) Aflavarin-411; The MS/MS spectra were acquired in IDA (information dependent acquisition) mode using a CE (collision energy) of 35 V with a collision energy spread (CES) of 15 V.

Aflavarin (*[m+H]^+^/z* 455.1353, Δ=3.5 ppm) exhibits a characteristic MS/MS spectrum with prominent fragment ions at m/z 425, 413, 395, 379, 363, 349, 333 and 303, most of them arising by the successive loss of the hydroxymethyl group and other methoxy functionalities from the chemical scaffold of the parent ion (Spectrum A). The cleavage of methoxy groups in the fragmentation behavior of aflavarin and its analogues, is most likely mediated as a neutral loss in the form of formaldehyde (HCHO). Aflavarin-439 (*[m+H]^+^/z* 439.1404, Δ=3.8 ppm) represents a dehydroxylated analog of aflavarin, thus exhibting a 16 Da mass subtraction with respect to aflavarin. This 16 Da difference is also noticeable in other key MS/MS fragments of aflavarin-439 such as m/z 439(455), 407 (423), 397 (413) (Spectrum B). Aflavarin-425 (*[m+H]^+^/z* 425.1246, Δ=3.5 ppm) is actually a demethylated derivative of aflavarin 439 showing a 14 Da difference as compared to each other, with regard to both parent and fragment ions at m/z 425 (439), 393 (407), 383 (397) (Spectrum C). Similarly, aflavarin-411 (*[m+H]^+^/z* 411.1078, Δ=0.9 ppm), being a demethylated analogue of aflavarin-425, exerts the 14 Da difference as depicted through fragment ions at m/z 411 (425), 379 (393), 369 (383) (Spectrum D).

Figure S6. High resolution MS/MS spectra: A) Dehydroaflavarin; B) Aflavarin-441; C) Novel derivative (*m/z* 437).The MS/MS spectra were acquired in IDA (information dependent acquisition) mode using a CE (collision energy) of 35 V with a collision energy spread (CES) of 15 V.

Dehydroaflavarin (*[m+H]^+^/z* 453.1190, Δ=2.2 ppm) and aflavarin-441 (*[m+H]^+^/z* 441.1187, Δ=1.6 ppm) show characteristic MS/MS spectra of aflavarins (Spectra A and B). Spectrum C corresponds to another metabolite eluting just before aflavarin and found to belong to this class of metabolites. Accurate mass measurements revealed an exact mass at *[m+H]^+^/z* 437.1226 corresponding with a chemical formula of C_24_H_20_O_8_ (Δ=-1.1 ppm). This implies two protons less (e.g. an extra double bond) than aflavarin-439, a pattern that can also be noticed for other ion in the MS/MS spectrum i.e. *m/z* 405 (*m/z* 407 for aflavarin-439) and 377 (*m/z* 379 for aflavarin-439). However, keeping in mind the structure of aflavarin-439, there is no logical place for an additional double bond. In this case, a different arrangement of the structure or a seco-derivative could be the explanation for the existence of this molecule. Elution of bicoumarin metabolites in several retention times could be due to the existence of different regioisomeric forms.

Figure S7. High resolution MS/MS spectra: A) Oxyasparasone A; B) Asparasone A (ESI-); C) Dehydroxyasparasone A; D) Deacetylasparasone A (ESI-). The MS/MS spectra were acquired in IDA (information dependent acquisition) mode using a CE (collision energy) of 35 V with a collision energy spread (CES) of 15 V.

Oxyasparasone A (*[m+H]^+^/z* 375.0723, Δ=3.5 ppm) contains an additional hydroxyl group as compared to asparasone A, thus exhibiting a 16 Da mass difference. Its MS/MS spectrum (Spectrum B) indicates the loss of two consecutive water molecules (2 x 18 Da) from the aliphatic side chain, yielding two respective product ions at m/z 357 and 339 (Figure S7A). Afterwards, the fragment ion at m/z 339 ejects a 42 Da (C_2_H_2_O) moiety to generate the fragment ion at m/z 297. Further downstream, the total removal of the side chain, followed by some consecutive losses of CO (28 Da) from the anthraquinone core structure, generates typical fragments such as the ions at m/z 243 and 213. Asparasone A (*[m-H]^-^/z* 357.0617, Δ=3.5 ppm) shows a typical MS/MS spectrum reflecting its dehydroxylated chemical scaffold as compared to oxyasparasone A (Spectrum B). Dehydroxyasparasone A (*[m+H]^+^/z* 341.0669, Δ=3.8 ppm) is a dehydrated analogue of asparasone A, missing the hydroxyl functionality in the C1' position of the aliphatic side chain. Prominent fragment ions at m/z 299 and m/z 271 could be seen in its MS/MS spectrum, corresponding with partial or total loss of the side chain (Spectrum C). Deacetylasparasone A (*[m-H]^-^/z* 315.0511, Δ=2.4 ppm) shows a typical MS/MS spectrum with diagnostic fragments at m/z 297, 269 and 253 (Spectrum D).

Figure S8. High resolution MS/MS spectra: A) 4'(5')-Hydroxyasperentin; B) 6 (8)-O-Methylasperentin; C) 6,8-Dimethylcitreoisocoumarin; The MS/MS spectra were acquired in IDA (information dependent acquisition) mode using a CE (collision energy) of 35 V with a collision energy spread (CES) of 15 V.

The MS/MS spectrum of 4'(5')-hydroxyasperentin (*[m+H]^+^/z* 309.1335, Δ=0.6 ppm) shows two abundant fragment ions at m/z 291 and 273, corresponding to the successive loss of two water molecules (-2 x 18 Da) from the methyl-substituted tetrahydropyran ring system (Spectrum A). This tatrahydropyran moiety underwent further fragmentation with sequential losses of 24, 52, 68 and 82 mass units to generate characteristic fragment ions at m/z 249, 221, 205 and 191, respectively. Similarly, 6(8)-*O*-methylasperentin (correctly named as 3(5)-*O*-methylasperentin; *[m+H]^+^/z* 307.1549, Δ=2.9 ppm) and with 4'(5')-hydroxyasperentin (eluting close to each other) demonstrate an analogical mode of fragmentation (Spectrum B). 6,8-Dimethylcitreoisocoumarin (correctly named as 3,5-dimethylcitreoisocoumarin; *[m+H]^+^/z* 307.1176, Δ<0.1 ppm) contains a 2'-hydroxy-4'-oxo-pentanyl side chain attached to the C2 position of the citreoisocoumarin nucleus. As with the other isocoumarins, the fragmentation events mainly occur in the side chain of the molecule with abundant ions at m/z 289, 247 and 221, corresponding with loss of a water molecule (- 18 Da) and other sequential cleavages of the 4'-oxo-pentenyl side chain (Spectrum C).

Figure S9. High resolution MS/MS spectra: A) Kojic acid; B) 7-*O*-Acetylkojic acid; C) Orsellinic acid (ESI-). The MS/MS spectra were acquired in IDA (information dependent acquisition) mode using a CE (collision energy) of 35 V with a collision energy spread (CES) of 15 V.

MS/MS spectrum of kojic acid shows a prominent parent ion at *[m+H]^+^/z* 143.0337 (Δ<0.1 ppm) and typical fragment ions at m/z 125, 97 and 69 that are characteristic for this metabolite (Spectrum A). The MS/MS spectrum of its acetylated derivative, i.e. 7-*O*-acetyl-kojic acid, (*[m+H]^+^/z* 185.0438, Δ=-3.2 ppm) shows a fragment at m/z 167, corresponding to an initial the loss of 18 mass units, while the rest of the fragments (i.e. fragments at m/z 143, 125, 97 and 69) are identical to those observed for kojic acid (Spectrum B).

Figure S10. High resolution MS/MS spectra: A) Aspergillic acid; B) Neoaspergillic acid; C) (Neo) Hydroxyaspergillic acid; D) Flavacol. The MS/MS spectra were acquired in IDA (information dependent acquisition) mode using a CE (collision energy) of 35 V with a collision energy spread (CES) of 15 V.

Aspergillic acid and neoaspergillic acid are structural isomers possessing identical chemical formulas and exact masses, the only difference being the position of one methyl group in the aliphatic side chain. This also holds for hydroxyaspergillic acid and neohydroxyaspergillic acid. Aspergillic acid eluted at RT 4.8 min with a protonated exact mass at *[m+H]^+^/z* 225.1604, (Δ= 1.1 ppm) and with prominent fragment peaks at m/z 207, 189, 165 and 137 (Spectrum A). A metabolite with the same monoisotopic mass and similar fragmentation behavior (Spectrum B) eluted at an earlier RT (4.6 min) and was assigned as neoaspergillic acid. Another metabolite, with a protonated exact mass at *[m+H]^+^/z* 241.1543 (Δ=- 1.53 ppm), that corresponds to hydroxy- or neohydroxy-aspergillic acid, eluted at RT 4.0 min with characteristic fragment ions at m/z 181, 153 and 100 (Spectrum C). Analogically, the dehydroxylated derivative of neo- or aspergillic acid, i.e. deoxyaspergillic acid or flavacol (*[m+H]^+^/z* 209.1649, Δ= 0.3 ppm), could be identified with its typical MS/MS spectrum (Spectrum D).

Figure S11. High resolution MS/MS spectra: A) 2,5-di-(p-hydroxybenzyl) piperazine; B) Actinopolymorphol C; C) Ditryptophenaline. The MS/MS spectra were acquired in IDA (information dependent acquisition) mode using a CE (collisionenergy) of 35 V with a collision energy spread (CES) of 15 V.

2,5-di-(p-hydroxybenzyl) piperazine (*[m+H]^+^/z* 299.1753, Δ=- 0.3 ppm) shows an MS/MS spectrum with characteristic fragment ions at m/z 211, 193, 162, 150 and 107 (Spectrum A). Fragment ions at m/z 193 and 107 result from the cleavage of the chemical bond between the C5 of the piperazine ring and methylene carbon of the p-hydroxybenzyl moiety. Ions at m/z 162 and 150 are a result of further fragmentation of the piperazine central ring. In the case of actinopolymorphol C (*[m+H]^+^/z* 293.1284, Δ< 0.1 ppm) two p-hydroxybenzyl moieties are linked via a pyrazine ring. Its MS/MS spectrum underwent a similar fragmentation with piperazines (Spectrum S11).

The MS/MS spectrum of ditryptophenaline shows the protonated molecule with *[m+H]^+^/z* 693.3209 (Δ= 3.5 ppm) and clear fragment ions at m/z 346, 318, 255, 157 and 130 (Spectrum C). The most prominent fragment ion of ditryptophenaline, m/z 346 corresponds to the N-methylphenylalanyl-tryptophanyl diketopiperazine monomer after cleavage of the C3-C3' chemical bond within the collision cell. High-resolution mass measurements revealed that the fragment at mass 318 corresponds to the chemical formula of C_20_H_20_N_3_O^+^, exhibiting a 28 Da loss as compared with the diketopiperazine monomer. Based on chemical composition and mass difference, this fragment is generated from a diketopiperazine monomer, via a neutral loss of a keto-group as carbon monoxide (CO) from ring D. The fragment ion at m/z 255 has a chemical composition of C_14_H_13_N_3_O_2_·^+^, generated by cleavage of the C15-C17 chemical bond and the loss of the benzylic moiety (ring E). The ion at m/z 157 arises from the total removal of the diketopiperazine ring (ring D) from the monomeric substructure. The fragment ion at m/z 130 represents an indole nucleus of the molecule.

Figure S12. High resolution MS/MS spectra of: A) α-CPA; B) β-CPA; C) α-CPA imine; D) 2-oxoCPA; E) Speradine A. The MS/MS spectra were acquired in IDA (information dependent acquisition) mode using a CE (collision energy) of 35 V with a collision energy spread (CES) of 15 V.

The occurrences of typical and diagnostic fragments in the MS/MS spectra of α-CPA (*[m+H]^+^/z* 337.1536, Δ= -0,9ppm; m/z 281, 196, 182, 167, 154, 140, 130; Spectrum A), β-CPA (*[m+H]^+^/z* 339.1700, Δ= -0,9 ppm; m/z 283, 198, 168, 130; Spectrum B), α-CPA imine (*[m+H]^+^/z* 336.1707, Δ<0,1 ppm; m/z 319, 280, 196, 139, 130, Spectrum C), 2-oxoCPA (*[m+H]^+^/z* 353.1504, Δ= 0,8 ppm; m/z 335, 311, 212, 208, 146; Spectrum D) and speradine A (*[m+H]^+^/z* 367.1648, Δ= -1,0 ppm; m/z 349, 307, 266, 226, 182, 160; Spectrum E) are in accordance with a previous report (Uka et al. 2017).

Figure S13. High resolution MS/MS spectra of: A) Leporin A; B) Leporin B; C) Leporin C; D) 8-Demethyl-leporin C; E) Hydroxy-leporin B; F) Iron-trioxoleporin B. The MS/MS spectra were acquired in IDA (information dependent acquisition) mode using a CE (collision energy) of 35 V with a collision energy spread (CES) of 15 V.

Figure S14. High resolution MS/MS spectra of aflavinine-type of indole-diterpenoids: A) Aflavinine; B) 14-Hydroxyaflavinine; C) 14,25-Dihydroxyaflavinine. The MS/MS spectra were acquired in IDA (information dependent acquisition) mode using a CE (collision energy) of 35 V with a collision energy spread (CES) of 15 V.

Apart from the parent ion, the MS/MS spectrum of aflavinine (*[m+H]^+^/z* 406.3107, Δ=0,74 ppm) shows a prominent fragment at m/z 388, which is attributed to the loss of the hydroxyl functionality from the C19 of the ring E (Spectrum A). Another interesting ion at m/z 306 is generated after complete removal of ring E from the tricyclic-diterpenoid condensed system. Accurate mass measurements revealed that fragment ions at m/z 289, 271, 247, 229, 215 and 201 correspond to chemical formulas for C_20_H_33_O^+^, C_20_H_31_^+^, C_17_H_27_O^+^, C_17_H_25_^+^, C_16_H_23_^+^ and C_15_H_21_^+^ respectively, and all represent different variants of fragmentation of the diterpenoid moiety. This is supported by the fact that there are no nitrogen atoms as part of their chemical formulas. Ions at m/z 130 and 158 are typical representatives of the indole core structure. Analogically, the MS/MS spectrum of 14-hydroxyaflavinine (*[m+H]^+^/z* 422.3053, Δ<0,1 ppm) shows two prominent ions at m/z 404 and 386, both representing successive losses of functional hydroxyl groups from positions at C14 and C19 of the diterpenoid moiety (Spectrum B). Further fragmentation of monohydroxyaflavinine moiety occurs in a similar pattern as depicted for aflavinine. A similar mode of fragmentation was observed for 14,25-dihydroxyaflavinine ([m+H]+/z 438.3017, Δ=3.2 ppm) with three consecutive losses of hydroxyl functionalities, followed by the chronological fragmentation of the C_20_-terpenoid moiety (Spectrum C).

Figure S15. High resolution MS/MS spectra of aflatrem-type of indole-diterpenoids: A) Aflatrem; B) Hydroxyaflatrem. The MS/MS spectra were acquired in IDA(information dependent acquisition) mode using a CE (collision energy) of 35 V with a collisionenergy spread (CES) of 15 V.

The MS/MS spectrum of aflatrem (Spectrum A) shows a prominent fragment peak at m/z 198, representing the prenylated indole nucleus, which is a diagnostic ion for the whole group of prenylated indole-diterpenoids. Furthermore, this MS/MS spectrum showed other fragments at m/z 485 and 444, which are attributed to the gas phase destruction of ring G in the diterpenoid part of the core structure. More precisely, the ion at m/z 485 is more likely generated by the loss of the oxygen atom at position 14a as the hydroxyl radical, followed by the cleavage of the C15-linked isopropylic moiety to deliver the second fragment at m/z 444. Subsequently, the fragment ion at m/z 426 is generated from m/z 444 via loss of the C19-linked hydroxyl group as a water molecule. In addition, the ion at m/z 444 undergoes another path of fragmentation to generate a fragment peak at m/z 376 through cleavage of the dimethylallyl motif (C5-isoprene unit) attached to the indole moiety of the structure. Hydroxyaflatrem is an oxygenated analog of aflatrem. Its MS/MS data (Spectrum B) indicated that the hydroxyl group should be situated in the indole moiety of the core structure. The strongest argument supporting this idea is the prominence of the fragment ion at m/z 214, which actually represents the hydroxylated analogue of the ion at m/z 198 from the aflatrem MS/MS spectrum. Existence of ions at m/z 146 and 172 in the MS/MS spectrum of hydroxyaflatrem, which are hydroxylated analogs of ions at m/z 130 and 156, respectively, from the aflatrem spectrum excludes the possibility of hydroxyl attachments in the C5-dimethylallyl motif of the parent molecule. In this regard, the best options for location of this functional group are at positions C6, C7 or C8 of the indole nucleus, although we cannot exclude the C23 position of ring C.
