# Supplementary material for: Secondary Metabolite Dereplication and Phylogenetic Analysis Identify Various Emerging Mycotoxins and Reveal the High Intra-Species Diversity in Aspergillus flavus
Source: Front Microbiol. 2019 Apr 5;10:667. doi: 10.3389/fmicb.2019.00667 (PMC6461017; doi:10.3389/fmicb.2019.00667)
Supplement: Supplementary file 2 [file Data_Sheet_2.docx]

Table S1. Key fungal metabolites described in literature from different strains of *A. flavus*

| **Chemical class** | | **Name** | **Formula** | **Exact mass** | **[M+H^+^]** | **Reference** |
| --- | --- | --- | --- | --- | --- | --- |
| POLYKETIDES | Aflatoxins and precursors | Aflatoxin B_1_ | C17H12O6 | 312.06339 | 313.07066 | Yu et al., 2004; Hesseltine et al., 1966 |
|  |  | Aflatoxin B_2_ | C17H14O6 | 314.07904 | 315.08631 | Yu et al., 2004; Hesseltine et al., 1966 |
|  |  | Aflatoxin G_1_ | C17H12O7 | 328.0583 | 329.06558 | Yu et al., 2004; Hesseltine et al., 1966 |
|  |  | Aflatoxin G_2_ | C17H14O7 | 330.07395 | 331.08123 | Yu et al., 2004; Hesseltine et al., 1966 |
|  |  | DMST | C17H10O6 | 310.04774 | 311.05501 | Yu et al., 2004 |
|  |  | ST | C18H12O6 | 324.06339 | 325.07066 | Yu et al., 2004 |
|  |  | OMST | C19H14O6 | 338.07904 | 339.08631 | Yu et al., 2004 |
|  |  | DHDMST | C17H12O6 | 312.06339 | 313.07066 | Yu et al., 2004 |
|  |  | DHST | C18H14O6 | 326.07904 | 327.08631 | Yu et al., 2004 |
|  |  | DHOMST | C19H16O6 | 340.09469 | 341.10196 | Yu et al., 2004 |
|  |  | Aspertoxin | C19H14O7 | 354.07395 | 355.08123 | Rodricks et al., 1968 |
|  |  | AFOH | C17H14O6 | 314.07904 | 315.08631 | Nakazato et al., 1990 |
|  | Bicoumarins | Aflavarin | C24H22O9 | 454.12638 | 455.13366 | Cary et al., 2015a; TePaske et al., 1992 |
|  |  | Dehydroaflavarin | C24H20O9 | 452.11073 | 453.11801 | Cary et al., 2015a |
|  |  | Aflavarin-441 | C23H20O9 | 440.11073 | 441.11801 | Cary et al., 2015a |
|  |  | Aflavarin-439 | C24H22O8 | 438.13147 | 439.13874 | Cary et al., 2015a |
|  |  | Aflavarin-425 | C23H20O8 | 424.11582 | 425.12309 | Cary et al., 2015a |
|  |  | Aflavarin-411 | C22H18O8 | 410.10017 | 411.10744 | Cary et al., 2015a |
|  | Anthraquinones and anthrones/anthranols | Asparasone A | C18H14O8 | 358.06887 | 359.07614 | Cary et al., 2014 |
|  |  | Deacetylasparasone A | C16H12O7 | 316.0583 | 317.06558 | Cary et al., 2014 |
|  |  | Oxyasparasone A | C18H14O9 | 374.06378 | 375.07106 | Cary et al., 2014 |
|  |  | Dehydroxyasparasone A | C18H12O7 | 340.0583 | 341.06558 | Cary et al., 2014 |
|  |  | Asperflavin | C16H16O5 | 288.09977 | 289.10705 | Grove JF, 1972a |
|  |  | Anhydroasperflavin | C16H14O4 | 270.08921 | 271.09649 | Grove JF, 1972a |

Table S1. *Continued*

| **Chemical class** | | **Name** | **Formula** | **Exact mass** | **[M+H^+^]** | **Reference** |
| --- | --- | --- | --- | --- | --- | --- |
| POLYKETIDES | Small polyketides | Kojic Acid | C6H6O4 | 142.02661 | 143.03389 | Basappa et al., 1970 |
|  |  | 7-*O*-Acetylkojic acid | C8H8O5 | 184.03717 | 185.04445 | Sun et al., 2014 |
|  |  | Kojic acid dimer | C12H10O8 | 282.03756 | 283.04054 | Basappa et al., 1970; Sun et al., 2014 |
|  |  | 5-Hydroxymethylfuran-3-carboxylic acid | C6H6O4 | 142.0266 | 143.0339 | Ma et al., 2016 |
|  |  | 5-Acetoxymethylfuran-3-carboxylic acid | C8H8O5 | 184.0372 | 185.0444 | Ma et al., 2016 |
|  |  | 3-(p-Hydroxyphenyl)propandiol | C9H12O3 | 168.0786 | 169.0859 | Forseth et al., 2013 |
|  |  | 5,7-Dihydroxy-4-methylphthalide | C9H8O4 | 180.04226 | 181.04954 | Grove, 1972a |
|  |  | Asperfuran | C13H14O3 | 218.09429 | 219.10157 | Forseth et al., 2013 |
|  | Coumarins | Asperentin | C16H20O5 | 292.13107 | 293.13835 | Grove, 1972b |
|  |  | Asperentin 6-methyl ether | C17H22O5 | 306.14672 | 307.154 | Grove, 1972b |
|  |  | Asperentin 8-methyl ether | C17H22O5 | 306.14672 | 307.154 | Grove, 1972b |
|  |  | 4'-Hydroxyasperentin | C16H20O6 | 308.12599 | 309.13327 | Grove, 1973 |
|  |  | 5'-Hydroxyasperentin | C16H20O6 | 308.12599 | 309.13327 | Grove, 1972b |
|  |  | 8-*O*-Methyl-5'-hydroxyasperentin | C17H22O6 | 322.1416 | 323.1489 | Grove, 1973 |
|  |  | 6,8-Dimethylcitreoisocoumarin | C16H18O6 | 306.11034 | 307.11762 | Sun et al., 2014 |
| NON-RIBOSOMAL PEPTIDES | Pyrazines | Aspergillic acid | C12H20N2O2 | 224.15248 | 225.15975 | Assante et al., 1981 |
|  |  | Neoaspergillic acid | C12H20N2O2 | 224.15248 | 225.15975 | Assante et al., 1981 |
|  |  | Hydroxyaspergillic acid | C12H20N2O3 | 240.14739 | 241.15467 | Dutcher, 1958; Dutcher, 1947 |
|  |  | Neohydroxyaspergillic acid | C12H20N2O3 | 240.14739 | 241.15467 | Dutcher, 1958; Dutcher, 1947 |
|  |  | Mutaspergillic acid | C11H18N2O3 | 226.13174 | 227.13902 | Dutcher, 1958; Dutcher, 1947 |
|  |  | Flavacol | C12H20N2O | 208.15756 | 209.16484 | Dutcher, 1958; Dunn et al., 1949 |
|  |  | Hydroxyflavacol | C12H20N2O2 | 224.15248 | 225.15975 | Dutcher, 1958; Dutcher, 1947 |
|  |  | Ferriaspergillin | C36H57FeN6O6 | 725.3689 | 726.37617 | Assante et al., 1981 |
|  |  | Oximoaspergillimide | C11H20N2O3 | 228.14739 | 229.15467 | Assante et al., 1981 |
|  |  | Actinopolymorphol C | C18H16N2O2 | 292.12118 | 293.12845 | Forseth et al., 2013 |

Table S1. *Continued*

| **Chemical class** | | **Name** | **Formula** | **Exact mass** | **[M+H^+^]** | **Reference** |
| --- | --- | --- | --- | --- | --- | --- |
| NON-RIBOSOMAL PEPTIDES | Piperazines | cis-2,5-di-(p-hydroxybenzyl) piperazine | C18H22N2O2 | 298.16813 | 299.1754 | Forseth et al., 2013 |
|  |  | trans-2,5-di-(p-hydroxybenzyl) piperazine | C18H22N2O2 | 298.16813 | 299.1754 | Forseth et al., 2013 |
|  |  | 2-hydroxy-2,5-di-(p-hydroxybenzyl) morpholine | C18H21NO4 | 315.14706 | 316.15433 | Forseth et al., 2013 |
|  |  | 3-(p-hydroxybenzyl)  hexahydropyrrolo[1,2-a]  pyrazine-1,4-dione | C14H16N2O3 | 260.1161 | 261.1234 | Forseth et al., 2013 |
|  |  | Cyclo (D-N-methyl-Leu-L-Trp) | C18H23N3O2 | 313.1790 | 314.1863 | Klausmeyer et al., 2005 |
|  | Peptides | Miyakamides A_1_ | C31H32N4O3 | 508.24744 | 509.25472 | Shiomi et al., 2002 |
|  |  | Miyakamides B_1_ | C31H32N4O4 | 524.24236 | 525.24963 | Shiomi et al., 2002 |
|  |  | Miyakamides A_2_ | C31H32N4O3 | 508.24744 | 509.25472 | Shiomi et al., 2002 |
|  |  | Miyakamides B_2_ | C31H32N4O4 | 524.24236 | 525.24963 | Shiomi et al., 2002 |
|  | Diketo-piperazines | Ditryptophenaline | C42H40N6O4 | 692.3111 | 693.31838 | Saruwatari et al., 2014 |
|  |  | Aspirochlorine | C12H9ClN2O5S2 | 359.96414 | 360.97142 | Klausmeyer et al., 2005 |
|  |  | Trithioaspirochlorine | C12H9ClN2O5S3 | 391.93621 | 392.94349 | Klausmeyer et al., 2005 |
|  |  | Tetrathioaspirochlorine | C12H9ClN2O5S4 | 423.90829 | 424.91556 | Klausmeyer et al., 2005 |
|  |  | ß-Nitropropanoic Acid | C3H5NO4 | 119.02186 | 120.02913 | Bush et al., 1951 |
| PK-NRPs | Indole-tetramates | α-CPA | C20H20N2O3 | 336.14739 | 337.15467 | Luk et al., 1977 |
|  |  | β-CPA | C20H22N2O3 | 338.16304 | 339.17032 | Uka et al., 2017 |
|  |  | α-CPA imine | C20H21N3O2 | 335.16338 | 336.17065 | Uka et al., 2017 |
|  |  | 2-oxoCPA | C20H20N2O4 | 352.14231 | 353.14958 | Uka et al., 2017 |
|  |  | Speradine A | C21H22N2O4 | 366.15796 | 367.16523 | Uka et al., 2017 |
|  | 2-Pyridones | Leporin A | C23H27NO3 | 365.19909 | 366.20637 | Cary et al., 2015b |
|  |  | Leporin B | C22H25NO3 | 351.18344 | 352.19072 | Cary et al., 2015b |
|  |  | Leporin C | C22H25NO2 | 335.18853 | 336.19581 | Cary et al., 2015b |
|  |  | Fe Complex (Iron trioxoleporin B) | C66H72FeN3O9 | 1106.4618 | 1107.4690 | Cary et al., 2015b |

Table S1. *Continued*

| **Chemical class** | | **Name** | **Formula** | **Exact mass** | **[M+H^+^]** | **Reference** |
| --- | --- | --- | --- | --- | --- | --- |
| PK-NRPs | 2-Pyridones | 8-Demethyl-leporin C | C21H23NO2 | 321.17288 | 322.18016 | Arroyo-Manzanares et al., 2015 |
|  |  | 9,10-Dehydro-leporin C | C22H23NO2 | 333.1729 | 334.1820 | Cary et al., 2015b |
|  |  | 9,10-Dehydro-leporin B | C22H23NO3 | 349.1678 | 350.1751 | Cary et al., 2015b |
|  |  | 17-Hydroxy-14,15-dihydro-leporin C | C22H27NO3 | 353.1991 | 354.2064 | Arroyo-Manzanares et al., 2015 |
| INDOLE-DITERPENES | Aflavinines and derivatives | Aflavinine | C28H39NO | 405.30317 | 406.31044 | Gallagher et al., 1980; Nozawa et al., 1989 |
|  |  | Isoaflavinine | C28H39NO | 405.30317 | 406.31044 | Nozawa et al., 1989 |
|  |  | 14-Hydroxyaflavinine + epimer | C28H39NO2 | 421.29808 | 422.30536 | Nozawa et al., 1989 |
|  |  | 14-Hydroxyisoaflavinine + epimer | C28H39NO2 | 421.29808 | 422.30536 | Nozawa et al., 1989 |
|  |  | 14,25-Dihydroxyaflavinine + epi. | C28H39NO3 | 437.29299 | 438.30027 | Nozawa et al., 1989 |
|  |  | 21-oxo-isoaflavinine | C28H37NO2 | 419.28243 | 420.28971 | Nozawa et al., 1989 |
|  |  | 10,23,24,25-tetrahydro-24-hydroxyaflavinine | C28H41NO2 | 423.31373 | 424.32101 | Nozawa et al., 1989 |
|  |  | Aflavazole | C28H35NO2 | 417.26678 | 418.27406 | Tepaske et al., 1990 |
|  |  | Aflavinine-like metabolite | C28H37NO4 | 451.2723 | 452.2795 | Forseth et al., 2013 |
|  | Aflatrem and derivatives | Aflatrem | C32H39NO4 | 501.28791 | 502.29519 | Nicholson et al., 2009; TePaske et al., 1992 |
|  |  | Beta-aflatrem | C32H39NO4 | 501.28791 | 502.29519 | Nicholson et al., 2009; TePaske et al., 1992 |
|  |  | Hydroxyaflatrem | C32H39NO5 | 517.28282 | 518.2901 | Nicholson et al., 2009 |
|  |  | Paspaline | C28H39NO2 | 421.29808 | 422.30536 | Nicholson et al., 2009 |
|  |  | Paspalinine | C27H31NO4 | 433.22531 | 434.23259 | Nicholson et al., 2009 |
|  |  | Paspalicine | C27H31NO3 | 417.23039 | 418.23767 | Nicholson et al., 2009 |
|  |  | Paspalininol | C27H33NO4 | 435.24096 | 436.24824 | Nicholson et al., 2009 |
|  |  | Paxilline | C27H33NO4 | 435.24096 | 436.24824 | Nicholson et al., 2009 |
|  |  | PCM6 | C27H35NO3 | 421.26169 | 422.26897 | Nicholson et al., 2009 |
|  |  | 13-Desoxypaxilline | C27H33NO3 | 419.24604 | 420.25332 | Nicholson et al., 2009 |
| RiPPs | | Ustiloxin B | C26H39N5O12S | 645.23159 | 646.23887 | Umemura et al., 2014 |
|  |  | Ustiloxin F | C21H30N4O8 | 466.2064 | 467.2136 | Umemura et al., 2014 |

PK-NRPs (polyketide-non ribosomal peptides); RiPPs (ribosomally synthesized and post-translationally modified peptides)

**Table S2. Primers and PCR conditions used for molecular studies of 55 *A. flavus* strains**

| **Locus** | **Forward Primer** | **Reverse Primer** | **Cycle conditions^1^** | **Amplicon size (bp)** |
| --- | --- | --- | --- | --- |
| *amdS*^2^ | GCYGCRTTYAAYTCYTTRTAY | CTYACAACVYCATCRAAATC | 52.6 ^o^C; 1min; 40x | ~300 |
| *benA* | GGTAACCAAATCGGTGCTGCTTTC | ACCCTCAGTGTAGTGACCCTTGGC | 56 ^o^C; 45s; 33x | ~500 |
| *cmdA*^2^ | CCGAGTACAAGGAGGCCTTC | TTTYTGCATCATRAGYTGGAC | 51 ^o^C; 45s; 33x | ~700 |
| *trpC*^2^ | TTTTGGCTGARGTCAACAWTC | ACKAAGACCTGGAKVTRAGA | 52.6 ^o^C; 1min; 40x | ~400 |
| *aflF/aflU* | GTGCCCAGCATCTTGGTCCA | AGGACTTGATGATTCCTCGTC | 55 ^o^C; 2min; 33x | ~200-1800 |
| *MAT1-1^3^* | ATGGAAACCACAGTGTCTCC | TCAACGAATCTAGAGAAGTC | 58 ^o^C; 1.15min; 33x | ~1000 |
| *MAT1-2^3^* | GCATTCATCCTTTATCGTCAGC | GCTTCTTTTCGGATGGCTTGCG | 58 ^o^C; 1.15min; 33x | ~300 |

^1^ Cycle conditions include annealing temperature (30s); extension time (72^o^C); and number of cycles

^2^ Indicates use of degenerate primers

^3^ MAT primers were pooled into a single reaction for diagnostic PCR. Only a portion of MAT1-2 was amplified for ease of differentiation between MAT genes.

**Table S3. Polyketide metabolites detected in our study of 55 *A. flavus* isolates**

| ***S*trains**  **(SRRC)** | **Polyketides** | | | | | | | | | | | | | | | | | | | | | | | | | | | | | |  |  |  |
| --- | --- | --- | --- | --- | --- | --- | --- | --- | --- | --- | --- | --- | --- | --- | --- | --- | --- | --- | --- | --- | --- | --- | --- | --- | --- | --- | --- | --- | --- | --- | --- | --- | --- |
|  | **Aflatoxins and precursors** | | | | | | | | | | | | **Bicoumarins** | | | | | | | **Coumarins/Anthraquinones/Small polyketides** | | | | | | | | | | |  |  |  |
|  | **Aflatoxin B1** | **Aflatoxin B2** | **Aflatoxin G1** | **Aflatoxin G2** | **Aflatoxin M1** | **Aflatoxin M2** | **Aflatoxin der. 331** | **ST** | **OMST** | **DHST** | **DHOMST** | **Aspertoxin** | | **Aflavarin (AFV)** | **Dehydroaflavarin** | **7-*O*-Demethyl-AFV** | **3,8'-Bisiderin** | **AFV variant 425** | **AFV variant 411** | **Asparasone A** | **Asparasone 317** | | **Asparasone 375** | **Asparasone 341** | **Orsellinic acid** | **Kojic acid (KA)** | **7-*O*-Acetyl KA** | **Asperentin-6 (8)- methyl ether** | **4'(5')-Hydroxy**  **asperentin** | **6,8-Dimethylcitreo**  **isocoumarin** |  | | |
| **38** | **-** | **-** | **-** | **-** | **-** | **-** | **-** | **-** | **-** | **-** | **-** | **-** | | **++** | **-** | **-** | **++** | **++** | **++** | **++** | | **+** | **+** | **+** | **+** | **-** | **-** | **-** | **-** | **+** |  | |  |
| **141** | **+** | **++** | **-** | **-** | **+** | **+** | **+** | **-** | **-** | **+** | **++** | **-** | | **-** | **-** | **-** | **-** | **-** | **-** | **+** | | **+** | **++** | **++** | **+** | **-** | **-** | **+** | **-** | **-** |  | |  |
| **144** | **++** | **-** | **-** | **-** | **-** | **-** | **-** | **-** | **+** | **-** | **-** | **+** | | **+** | **-** | **-** | **+** | **+** | **+** | **+** | | **+** | **++** | **+** | **-** | **-** | **-** | **-** | **-** | **++** |  | |  |
| **150** | **-** | **-** | **-** | **-** | **-** | **-** | **-** | **-** | **-** | **-** | **-** | **-** | | **-** | **-** | **-** | **-** | **-** | **-** | **-** | | **-** | **-** | **-** | **-** | **-** | **-** | **-** | **+** | **++** |  | |  |
| **151** | **-** | **-** | **-** | **-** | **-** | **-** | **-** | **-** | **+** | **-** | **-** | **-** | | **-** | **-** | **-** | **-** | **-** | **-** | **-** | | **-** | **-** | **-** | **-** | **-** | **-** | **-** | **-** | **++** |  | |  |
| **167** | **++** | **++** | **-** | **-** | **++** | **+** | **++** | **+** | **++** | **-** | **-** | **+** | | **++** | **-** | **-** | **+** | **+** | **+** | **+** | | **+** | **++** | **+** | **-** | **-** | **-** | **-** | **-** | **-** |  | |  |
| **283** | **-** | **-** | **-** | **-** | **-** | **-** | **-** | **-** | **-** | **-** | **-** | **-** | | **-** | **-** | **-** | **-** | **-** | **-** | **+** | | **+** | **++** | **+** | **+** | **-** | **-** | **-** | **-** | **-** |  | |  |
| **295** | **+** | **-** | **-** | **-** | **-** | **-** | **-** | **-** | **-** | **-** | **-** | **-** | | **-** | **-** | **-** | **-** | **-** | **-** | **++** | | **++** | **+** | **+** | **+** | **-** | **-** | **-** | **-** | **-** |  | |  |
| **1000F** | **++** | **++** | **-** | **-** | **++** | **+** | **+** | **-** | **++** | **-** | **++** | **++** | | **+** | **-** | **-** | **-** | **-** | **+** | **+** | | **-** | **++** | **++** | **-** | **-** | **-** | **-** | **-** | **-** |  | |  |
| **1006** | **-** | **-** | **-** | **-** | **-** | **-** | **-** | **-** | **-** | **-** | **-** | **-** | | **++** | **-** | **-** | **+** | **+** | **+** | **+** | | **+** | **+** | **+** | **+** | **-** | **-** | **++** | **-** | **-** |  | |  |
| **1020** | **-** | **-** | **-** | **-** | **-** | **-** | **-** | **-** | **-** | **-** | **-** | **+** | | **++** | **+** | **+** | **++** | **++** | **++** | **+** | | **+** | **+** | **+** | **+** | **-** | **-** | **-** | **-** | **-** |  | |  |
| **1021** | **-** | **-** | **-** | **-** | **-** | **-** | **-** | **-** | **-** | **-** | **-** | **-** | | **-** | **-** | **-** | **-** | **-** | **-** | **-** | | **-** | **-** | **-** | **-** | **+** | **+** | **-** | **-** | **++** |  | |  |
| **1055** | **++** | **++** | **-** | **-** | **++** | **+** | **++** | **-** | **++** | **-** | **+** | **++** | | **+** | **+** | **+** | **++** | **++** | **++** | **+** | | **+** | **++** | **++** | **-** | **-** | **-** | **-** | **-** | **+** |  | |  |
| **1071** | **++** | **+** | **-** | **-** | **+** | **-** | **-** | **-** | **-** | **-** | **-** | **+** | | **-** | **-** | **-** | **-** | **-** | **-** | **-** | | **-** | **-** | **-** | **-** | **-** | **-** | **-** | **-** | **-** |  | |  |
| **1098** | **-** | **-** | **-** | **-** | **-** | **-** | **-** | **-** | **-** | **-** | **-** | **+** | | **+** | **-** | **-** | **++** | **++** | **++** | **+** | | **-** | **++** | **++** | **-** | **-** | **-** | **-** | **++** | **+** |  | |  |
| **1118** | **++** | **++** | **++** | **++** | **+** | **+** | **+** | **++** | **++** | **+** | **-** | **++** | | **-** | **-** | **-** | **+** | **+** | **+** | **++** | | **++** | **+** | **+** | **+** | **-** | **-** | **-** | **+** | **-** |  | |  |
| **1187** | **++** | **-** | **-** | **-** | **-** | **-** | **-** | **-** | **+** | **++** | **-** | **+** | | **++** | **-** | **-** | **+** | **+** | **+** | **+** | | **-** | **++** | **++** | **+** | **-** | **-** | **-** | **++** | **+** |  | |  |
| **1299** | **++** | **++** | **-** | **-** | **++** | **-** | **++** | **-** | **+** | **-** | **+** | **+** | | **++** | **-** | **+** | **++** | **++** | **++** | **+** | | **-** | **++** | **++** | **-** | **-** | **-** | **+** | **-** | **-** |  | |  |
| **1356** | **-** | **-** | **-** | **-** | **-** | **-** | **-** | **-** | **-** | **-** | **-** | **-** | | **-** | **-** | **-** | **-** | **-** | **-** | **-** | | **-** | **-** | **-** | **-** | **-** | **-** | **-** | **+** | **+** |  | |  |
| **1357** | **-** | **-** | **-** | **-** | **-** | **-** | **-** | **-** | **-** | **-** | **-** | **-** | | **-** | **-** | **-** | **-** | **-** | **-** | **-** | | **-** | **-** | **-** | **-** | **-** | **-** | **-** | **++** | **++** |  | |  |
| **1533** | **-** | **-** | **-** | **-** | **-** | **-** | **-** | **-** | **-** | **-** | **-** | **-** | | **-** | **-** | **-** | **-** | **-** | **-** | **-** | | **-** | **-** | **-** | **-** | **-** | **-** | **-** | **+** | **++** |  | |  |
| **1534** | **-** | **-** | **-** | **-** | **-** | **-** | **-** | **-** | **-** | **-** | **-** | **-** | | **++** | **+** | **+** | **++** | **++** | **++** | **++** | | **+** | **++** | **+** | **+** | **-** | **+** | **-** | **+** | **+** |  | |  |
| **1540** | **-** | **-** | **-** | **-** | **-** | **-** | **-** | **-** | **-** | **-** | **-** | **-** | | **+** | **-** | **-** | **-** | **-** | **-** | **+** | | **-** | **+** | **-** | **-** | **-** | **-** | **-** | **+** | **++** |  | |  |
| **1541** | **-** | **-** | **-** | **-** | **-** | **-** | **-** | **-** | **-** | **-** | **-** | **-** | | **-** | **-** | **-** | **-** | **-** | **-** | **-** | | **-** | **-** | **-** | **+** | **-** | **-** | **-** | **-** | **-** |  | |  |
| **1543** | **-** | **-** | **-** | **-** | **-** | **-** | **-** | **-** | **-** | **-** | **-** | **-** | | **-** | **-** | **-** | **-** | **-** | **-** | **-** | | **-** | **-** | **-** | **-** | **-** | **-** | **-** | **-** | **+** |  |  |  |
| **1544** | **-** | **-** | **-** | **-** | **-** | **-** | **-** | **-** | **-** | **-** | **-** | **-** | | **-** | **-** | **-** | **-** | **-** | **-** | **-** | | **-** | **-** | **-** | **-** | **-** | **-** | **-** | **+** | **+** |  | | |
| **1545** | **-** | **-** | **-** | **-** | **-** | **-** | **-** | **-** | **-** | **-** | **-** | **+** | | **++** | **+** | **+** | **+** | **+** | **++** | **+** | | **+** | **++** | **++** | **-** | **-** | **-** | **++** | **-** | **-** |  |  |  |
| **1547** | **-** | **-** | **-** | **-** | **-** | **-** | **-** | **-** | **-** | **-** | **-** | **-** | | **-** | **-** | **-** | **-** | **-** | **-** | **+** | | **+** | **+** | **++** | **+** | **-** | **-** | **-** | **++** | **++** |  |  |  |
| **1552** | **+** | **-** | **-** | **-** | **-** | **-** | **-** | **-** | **-** | **-** | **-** | **-** | | **-** | **-** | **-** | **-** | **-** | **-** | **+** | | **-** | **+** | **+** | **-** | **-** | **-** | **-** | **-** | **++** |  |  |  |
| **1553** | **-** | **-** | **-** | **-** | **-** | **-** | **-** | **-** | **-** | **-** | **-** | **+** | | **++** | **-** | **-** | **+** | **+** | **-** | **+** | | **+** | **++** | **+** | **-** | **-** | **-** | **-** | **++** | **++** |  |  |  |
| **1554** | **++** | **-** | **-** | **-** | **-** | **-** | **-** | **-** | **-** | **-** | **-** | **+** | | **++** | **+** | **+** | **++** | **++** | **++** | **++** | | **++** | **++** | **++** | **+** | **-** | **-** | **-** | **++** | **+** |  |  |  |
| **1557** | **-** | **-** | **-** | **-** | **-** | **-** | **-** | **-** | **-** | **-** | **-** | **-** | | **++** | **+** | **+** | **++** | **++** | **++** | **+** | | **-** | **++** | **-** | **-** | **-** | **-** | **-** | **-** | **-** |  |  |  |
| **1558** | **++** | **+** | **-** | **-** | **+** | **-** | **+** | **-** | **+** | **-** | **-** | **+** | | **-** | **-** | **-** | **-** | **-** | **-** | **+** | | **-** | **+** | **+** | **-** | **-** | **-** | **+** | **-** | **+** |  |  |  |
| **1559** | **++** | **-** | **-** | **-** | **-** | **-** | **-** | **-** | **-** | **-** | **-** | **+** | | **++** | **+** | **+** | **++** | **++** | **++** | **+** | | **-** | **+** | **+** | **-** | **-** | **-** | **-** | **++** | **+** |  |  |  |
| **1565** | **++** | **++** | **-** | **-** | **+** | **+** | **++** | **-** | **++** | **+** | **++** | **++** | | **++** | **-** | **-** | **+** | **+** | **+** | **++** | | **+** | **++** | **++** | **-** | **-** | **-** | **++** | **-** | **-** |  |  |  |
| **1566** | **++** | **++** | **-** | **-** | **++** | **+** | **++** | **+** | **+** | **+** | **+** | **+** | | **++** | **+** | **+** | **++** | **++** | **++** | **++** | | **+** | **++** | **++** | **+** | **-** | **-** | **-** | **-** | **-** |  |  |  |
| **1568** | **++** | **-** | **-** | **-** | **-** | **-** | **-** | **-** | **-** | **-** | **-** | **-** | | **-** | **-** | **-** | **-** | **-** | **-** | **-** | | **-** | **-** | **-** | **-** | **-** | **-** | **-** | **-** | **-** |  |  |  |
| **1571** | **-** | **-** | **-** | **-** | **-** | **-** | **-** | **-** | **-** | **-** | **-** | **-** | | **-** | **-** | **-** | **-** | **-** | **-** | **-** | | **-** | **-** | **-** | **+** | **-** | **-** | **-** | **-** | **++** |  |  |  |
| **1573** | **++** | **+** | **-** | **-** | **-** | **-** | **-** | **+** | **-** | **-** | **-** | **-** | | **-** | **-** | **-** | **-** | **-** | **-** | **++** | | **+** | **++** | **++** | **+** | **-** | **-** | **++** | **-** | **+** |  |  |  |
| **1574** | **-** | **-** | **-** | **-** | **-** | **-** | **-** | **-** | **-** | **-** | **-** | **-** | | **-** | **-** | **-** | **-** | **-** | **-** | **+** | | **-** | **+** | **+** | **-** | **-** | **-** | **-** | **+** | **+** |  |  |  |
| **1575** | **-** | **-** | **-** | **-** | **-** | **-** | **-** | **-** | **-** | **-** | **-** | **-** | | **-** | **-** | **-** | **-** | **-** | **-** | **-** | | **-** | **-** | **-** | **-** | **-** | **-** | **-** | **++** | **-** |  |  |  |
| **1576** | **++** | **++** | **-** | **-** | **++** | **-** | **++** | **-** | **+** | **-** | **+** | **++** | | **++** | **-** | **+** | **++** | **++** | **++** | **++** | | **++** | **++** | **+** | **+** | **-** | **-** | **-** | **-** | **-** |  |  |  |
| **1578** | **++** | **++** | **-** | **-** | **++** | **-** | **++** | **-** | **+** | **-** | **-** | **+** | | **++** | **+** | **+** | **++** | **++** | **++** | **+** | | **+** | **++** | **+** | **+** | **+** | **+** | **-** | **-** | **-** |  |  |  |
| **1591** | **++** | **++** | **++** | **++** | **+** | **-** | **+** | **-** | **++** | **-** | **+** | **++** | | **++** | **-** | **-** | **++** | **++** | **++** | **+** | | **++** | **+** | **+** | **+** | **-** | **-** | **+** | **-** | **+** |  |  |  |
| **1626** | **-** | **-** | **-** | **-** | **-** | **-** | **-** | **-** | **-** | **-** | **-** | **-** | | **-** | **-** | **-** | **-** | **-** | **-** | **-** | | **-** | **-** | **-** | **-** | **-** | **-** | **++** | **-** | **+** |  |  |  |
| **1637** | **-** | **-** | **-** | **-** | **-** | **-** | **-** | **-** | **-** | **-** | **-** | **-** | | **-** | **-** | **-** | **-** | **-** | **-** | **-** | | **-** | **-** | **-** | **+** | **-** | **-** | **-** | **++** | **-** |  |  |  |
| **2000** | **+** | **-** | **-** | **-** | **-** | **-** | **-** | **-** | **-** | **-** | **-** | **-** | | **++** | **-** | **-** | **++** | **++** | **+** | **++** | | **+** | **++** | **+** | **-** | **-** | **-** | **-** | **++** | **++** |  |  |  |
| **2001** | **++** | **-** | **-** | **-** | **-** | **-** | **+** | **-** | **+** | **-** | **-** | **+** | | **+** | **-** | **-** | **-** | **-** | **-** | **+** | | **+** | **+** | **+** | **+** | **+** | **+** | **-** | **+** | **++** |  |  |  |
| **2033** | **++** | **+** | **++** | **+** | **+** | **-** | **+** | **+** | **++** | **+** | **-** | **++** | | **-** | **-** | **-** | **-** | **-** | **-** | **+** | | **++** | **++** | **-** | **-** | **-** | **-** | **-** | **+** | **-** |  |  |  |
| **2035** | **++** | **++** | **-** | **-** | **++** | **+** | **++** | **-** | **++** | **-** | **+** | **++** | | **++** | **-** | **-** | **+** | **+** | **+** | **++** | | **+** | **+** | **+** | **-** | **-** | **-** | **-** | **-** | **-** |  |  |  |
| **2111** | **++** | **++** | **-** | **-** | **++** | **-** | **++** | **+** | **+** | **-** | **-** | **+** | | **++** | **+** | **+** | **++** | **++** | **++** | **+** | | **-** | **++** | **+** | **+** | **+** | **+** | **-** | **-** | **-** |  |  |  |
| **2114** | **++** | **++** | **++** | **++** | **+** | **-** | **+** | **-** | **++** | **-** | **++** | **++** | | **-** | **-** | **-** | **-** | **-** | **-** | **+** | | **-** | **+** | **+** | **-** | **-** | **-** | **-** | **+** | **-** |  |  |  |
| **2115** | **-** | **-** | **-** | **-** | **-** | **-** | **-** | **-** | **-** | **-** | **-** | **-** | | **+** | **-** | **-** | **+** | **+** | **+** | **+** | | **+** | **+** | **+** | **-** | **-** | **-** | **-** | **+** | **+** |  |  |  |
| **2118** | **++** | **-** | **-** | **-** | **-** | **-** | **-** | **-** | **-** | **-** | **-** | **-** | | **-** | **-** | **-** | **-** | **-** | **-** | **-** | | **-** | **-** | **-** | **-** | **-** | **-** | **-** | **+** | **+** |  |  |  |
| **2524** | **-** | **-** | **-** | **-** | **-** | **-** | **-** | **-** | **-** | **-** | **-** | **-** | | **++** | **+** | **+** | **++** | **++** | **+** | **+** | | **+** | **++** | **+** | **-** | **-** | **-** | **-** | **-** | **+** |  |  |  |
| **2111** | **++** | **++** | **-** | **-** | **++** | **-** | **++** | **+** | **+** | **-** | **-** | **+** | | **++** | **+** | **+** | **++** | **++** | **++** | **+** | | **-** | **++** | **+** | **+** | **+** | **+** | **-** | **-** | **-** |  |  |  |

"+", Peak area ≤10^4^; "++", Peak area>10^4^; "-" not detected; SRRC- Southern Regional Research Center

**Table S4. Nitrogen-containing metabolites detected in our study of 55 *A. flavus* isolates**

| ***S*trains**  **(SRRC)** | **Non-ribosomal peptides and hybrid molecules (PK-NRPs)** | | | | | | | | | | | | | | | | | | |  |
| --- | --- | --- | --- | --- | --- | --- | --- | --- | --- | --- | --- | --- | --- | --- | --- | --- | --- | --- | --- | --- |
|  | **NRPs** | | | | | | | | **PK-NRPs** | | | | | | | | | | |  |
|  | **Aspergillic acid** | **Neoaspergillic acid** | **Hydroxy-aspergillic acid** | **Neohydroxy-**  **aspergillic acid** | **Flavacol** | **Actinopolymorphol C** | **2,5-di(p-hydroxybenzyl) piperazine** | **Ditryptophenaline** | **α-CPA** | **β-CPA** | **α-CPA imine** | **2-oxoCPA** | **Speradine A** | **Leporin A** | **Leporin B** | **Leporin C** | **Iron trioxoleporin B** | **8-Demethyl-leporin C** | **Leporin derivative 370** |  |
| **38** | **-** | **-** | **-** | **-** | **-** | **+** | **-** | **++** | **++** | **++** | **++** | **++** | **+** | **-** | **+** | **+** | **-** | **-** | **-** |  |
| **141** | **-** | **-** | **-** | **-** | **-** | **-** | **-** | **++** | **+** | **-** | **-** | **+** | **++** | **-** | **+** | **+** | **-** | **-** | **-** |  |
| **144** | **++** | **++** | **-** | **-** | **+** | **+** | **-** | **++** | **++** | **++** | **-** | **++** | **+** | **-** | **+** | **++** | **-** | **-** | **-** |  |
| **150** | **++** | **++** | **-** | **-** | **+** | **++** | **-** | **++** | **++** | **++** | **++** | **++** | **+** | **-** | **++** | **+** | **-** | **-** | **+** |  |
| **151** | **-** | **-** | **-** | **-** | **-** | **-** | **-** | **++** | **-** | **-** | **-** | **-** | **-** | **-** | **++** | **+** | **+** | **-** | **-** |  |
| **167** | **+** | **+** | **-** | **-** | **-** | **-** | **-** | **++** | **++** | **++** | **+** | **++** | **-** | **-** | **-** | **++** | **-** | **-** | **-** |  |
| **283** | **+** | **+** | **-** | **-** | **+** | **+** | **-** | **++** | **++** | **++** | **-** | **++** | **++** | **-** | **++** | **+** | **-** | **-** | **-** |  |
| **295** | **+** | **+** | **-** | **-** | **+** | **-** | **-** | **-** | **++** | **++** | **-** | **++** | **++** | **-** | **-** | **-** | **-** | **-** | **-** |  |
| **1000F** | **+** | **+** | **-** | **-** | **-** | **-** | **-** | **++** | **++** | **++** | **-** | **++** | **-** | **-** | **++** | **+** | **-** | **-** | **+** |  |
| **1006** | **-** | **-** | **-** | **-** | **-** | **-** | **-** | **++** | **++** | **++** | **++** | **+** | **++** | **-** | **+** | **+** | **-** | **-** | **-** |  |
| **1020** | **-** | **-** | **-** | **-** | **-** | **++** | **-** | **++** | **++** | **++** | **++** | **+** | **-** | **-** | **++** | **+** | **++** | **-** | **-** |  |
| **1021** | **++** | **++** | **-** | **-** | **+** | **-** | **-** | **++** | **++** | **++** | **+** | **++** | **-** | **-** | **++** | **++** | **-** | **-** | **+** |  |
| **1055** | **+** | **+** | **-** | **-** | **-** | **-** | **-** | **++** | **++** | **+** | **+** | **++** | **-** | **-** | **++** | **++** | **++** | **-** | **-** |  |
| **1071** | **-** | **-** | **-** | **-** | **-** | **-** | **-** | **++** | **++** | **++** | **-** | **+** | **-** | **-** | **++** | **-** | **++** | **-** | **-** |  |
| **1098** | **-** | **-** | **-** | **-** | **-** | **+** | **-** | **++** | **++** | **+** | **-** | **-** | **-** | **-** | **++** | **+** | **+** | **-** | **-** |  |
| **1118** | **-** | **-** | **-** | **-** | **-** | **-** | **-** | **-** | **-** | **-** | **-** | **-** | **-** | **-** | **-** | **-** | **-** | **-** | **-** |  |
| **1187** | **+** | **+** | **-** | **-** | **-** | **++** | **-** | **++** | **++** | **++** | **-** | **++** | **-** | **-** | **++** | **+** | **-** | **-** | **-** |  |
| **1299** | **++** | **++** | **-** | **-** | **++** | **-** | **-** | **+** | **++** | **++** | **++** | **++** | **-** | **-** | **+** | **++** | **-** | **-** | **+** |  |
| **1356** | **+** | **+** | **-** | **-** | **+** | **-** | **-** | **++** | **++** | **-** | **++** | **++** | **++** | **-** | **+** | **+** | **-** | **-** | **+** |  |
| **1357** | **+** | **+** | **-** | **-** | **+** | **-** | **-** | **++** | **++** | **++** | **-** | **+** | **++** | **-** | **-** | **-** | **-** | **-** | **-** |  |
| **1533** | **+** | **+** | **-** | **-** | **-** | **-** | **-** | **++** | **++** | **++** | **-** | **++** | **-** | **-** | **++** | **+** | **+** | **-** | **-** |  |
| **1534** | **+** | **+** | **+** | **+** | **+** | **++** | **-** | **++** | **++** | **++** | **-** | **-** | **-** | **-** | **++** | **+** | **-** | **-** | **-** |  |
| **1540** | **-** | **-** | **++** | **++** | **-** | **-** | **-** | **++** | **++** | **+** | **-** | **++** | **++** | **-** | **++** | **++** | **++** | **-** | **-** |  |
| **1541** | **++** | **++** | **++** | **++** | **-** | **-** | **-** | **-** | **++** | **-** | **-** | **-** | **-** | **-** | **++** | **++** | **+** | **-** | **-** |  |
| **1543** | **-** | **-** | **-** | **-** | **-** | **+** | **-** | **++** | **++** | **-** | **-** | **-** | **++** | **-** | **++** | **+** | **+** | **-** | **-** |  |
| **1544** | **-** | **-** | **-** | **-** | **-** | **-** | **-** | **-** | **++** | **++** | **-** | **++** | **++** | **-** | **+** | **+** | **-** | **-** | **-** |  |
| **1545** | **-** | **-** | **+** | **+** | **-** | **+** | **-** | **++** | **++** | **++** | **++** | **++** | **-** | **-** | **+** | **-** | **-** | **-** | **-** |  |
| **1547** | **-** | **-** | **-** | **-** | **-** | **-** | **-** | **++** | **++** | **++** | **-** | **-** | **++** | **-** | **++** | **+** | **++** | **-** | **-** |  |
| **1552** | **-** | **-** | **-** | **-** | **-** | **-** | **-** | **++** | **++** | **-** | **-** | **+** | **++** | **-** | **+** | **+** | **-** | **-** | **-** |  |
| **1553** | **+** | **+** | **+** | **+** | **-** | **-** | **-** | **++** | **++** | **+** | **+** | **++** | **-** | **-** | **+** | **+** | **-** | **-** | **-** |  |
| **1554** | **-** | **-** | **-** | **-** | **-** | **+** | **-** | **++** | **++** | **+** | **+** | **++** | **+** | **-** | **+** | **++** | **-** | **-** | **-** |  |
| **1557** | **-** | **-** | **-** | **-** | **-** | **-** | **-** | **++** | **++** | **++** | **-** | **+** | **++** | **-** | **++** | **+** | **++** | **-** | **-** |  |
| **1558** | **+** | **+** | **+** | **+** | **-** | **-** | **-** | **++** | **++** | **++** | **-** | **++** | **-** | **-** | **++** | **+** | **-** | **-** | **+** |  |
| **1559** | **-** | **-** | **-** | **-** | **-** | **+** | **-** | **++** | **++** | **++** | **+** | **++** | **-** | **-** | **++** | **++** | **-** | **-** | **-** |  |
| **1565** | **++** | **+** | **+** | **-** | **-** | **-** | **-** | **++** | **++** | **++** | **-** | **+** | **-** | **-** | **++** | **++** | **++** | **-** | **-** |  |
| **1566** | **-** | **-** | **-** | **-** | **-** | **-** | **-** | **+** | **++** | **++** | **-** | **++** | **-** | **-** | **+** | **++** | **-** | **-** | **+** |  |
| **1568** | **+** | **+** | **+** | **+** | **-** | **-** | **-** | **++** | **-** | **-** | **-** | **-** | **-** | **-** | **++** | **+** | **-** | **-** | **-** |  |
| **1571** | **-** | **-** | **-** | **-** | **-** | **+** | **-** | **++** | **++** | **++** | **++** | **+** | **++** | **-** | **++** | **+** | **-** | **-** | **-** |  |
| **1573** | **+** | **+** | **-** | **-** | **+** | **++** | **-** | **++** | **++** | **++** | **-** | **+** | **-** | **-** | **+** | **++** | **-** | **-** | **++** |  |
| **1574** | **+** | **+** | **-** | **-** | **+** | **-** | **-** | **++** | **++** | **++** | **++** | **++** | **-** | **-** | **+** | **+** | **-** | **-** | **+** |  |
| **1575** | **-** | **-** | **-** | **-** | **-** | **-** | **-** | **++** | **++** | **++** | **-** | **-** | **++** | **-** | **+** | **+** | **-** | **-** | **+** |  |
| **1576** | **-** | **-** | **-** | **-** | **-** | **-** | **-** | **++** | **++** | **++** | **+** | **++** | **++** | **-** | **+** | **+** | **-** | **-** | **+** |  |
| **1578** | **+** | **+** | **-** | **-** | **-** | **-** | **-** | **++** | **++** | **++** | **++** | **++** | **-** | **-** | **++** | **++** | **+** | **-** | **-** |  |
| **1591** | **++** | **++** | **-** | **-** | **+** | **-** | **-** | **-** | **++** | **++** | **-** | **++** | **++** | **+** | **++** | **++** | **-** | **-** | **-** |  |
| **1626** | **-** | **-** | **-** | **-** | **-** | **-** | **-** | **++** | **++** | **++** | **+** | **++** | **++** | **-** | **+** | **+** | **-** | **-** | **-** |  |
| **1637** | **+** | **+** | **-** | **-** | **+** | **-** | **-** | **++** | **++** | **++** | **+** | **++** | **++** | **-** | **+** | **+** | **-** | **-** | **-** |  |
| **2000** | **-** | **-** | **-** | **-** | **-** | **-** | **-** | **++** | **++** | **-** | **-** | **++** | **-** | **-** | **+** | **+** | **-** | **-** | **-** |  |
| **2001** | **-** | **-** | **-** | **-** | **-** | **+** | **-** | **++** | **++** | **++** | **+** | **+** | **-** | **-** | **++** | **++** | **-** | **-** | **-** |  |
| **2033** | **-** | **-** | **-** | **-** | **-** | **-** | **-** | **-** | **++** | **++** | **++** | **++** | **-** | **-** | **++** | **+** | **-** | **-** | **+** |  |
| **2035** | **+** | **+** | **+** | **+** | **-** | **-** | **-** | **-** | **++** | **++** | **-** | **++** | **-** | **-** | **++** | **+** | **-** | **-** | **-** |  |
| **2114** | **-** | **-** | **-** | **-** | **-** | **-** | **-** | **-** | **-** | **-** | **-** | **-** | **-** | **-** | **-** | **-** | **-** | **-** | **-** |  |
| **2115** | **-** | **-** | **-** | **-** | **-** | **-** | **-** | **++** | **++** | **++** | **++** | **++** | **-** | **-** | **+** | **+** | **-** | **-** | **-** |  |
| **2118** | **++** | **++** | **-** | **-** | **++** | **-** | **-** | **++** | **++** | **-** | **++** | **++** | **+** | **-** | **++** | **++** | **-** | **+** | **+** |  |
| **2524** | **-** | **-** | **+** | **+** | **-** | **+** | **+** | **++** | **++** | **++** | **-** | **++** | **-** | **-** | **++** | **+** | **+** | **-** | **-** |  |
| **2111** | **-** | **-** | **-** | **-** | **-** | **-** | **-** | **+** | **++** | **-** | **-** | **++** | **-** | **-** | **++** | **++** | **++** | **-** | **+** |  |

"+", Peak area ≤10^4^; "++", Peak area>10^4^; "-" not detected; SRRC- Southern Regional Research Center

Table S5. Indole-diterpenoid metabolites detected in this study of 55 A. flavus isolates

| ***S*trains**  **(SRRC)** | **Indole-diterpenes** | | | | | | |  |
| --- | --- | --- | --- | --- | --- | --- | --- | --- |
|  | **Aflavinines** | | | | **Aflatrems** | | |  |
|  | **Aflavinine** | **14-Hydroxyaflavinine** | **14-Hydroxy**  **isoaflavinine** | **14,25-Dihydroxy**  **aflavinine** | **Aflatrem** | **Beta-aflatrem** | **Hydroxyaflatrem** |  |
| **038** | **++** | **++** | **++** | **++** | **++** | **++** | **-** |  |
| **141** | **+** | **+** | **+** | **+** | **++** | **+** | **-** |  |
| **144** | **++** | **+** | **+** | **++** | **++** | **++** | **-** |  |
| **150** | **+** | **+** | **+** | **+** | **++** | **+** | **-** |  |
| **151** | **-** | **-** | **-** | **-** | **-** | **-** | **-** |  |
| **167** | **+** | **++** | **++** | **++** | **++** | **-** | **-** |  |
| **283** | **+** | **+** | **+** | **+** | **+** | **+** | **-** |  |
| **295** | **++** | **++** | **++** | **++** | **++** | **++** | **-** |  |
| **1000F** | **-** | **-** | **-** | **-** | **+** | **+** | **-** |  |
| **1006** | **++** | **++** | **++** | **++** | **++** | **++** | **+** |  |
| **1020** | **++** | **++** | **++** | **++** | **++** | **++** | **-** |  |
| **1021** | **-** | **-** | **-** | **-** | **-** | **-** | **-** |  |
| **1055** | **++** | **++** | **++** | **++** | **++** | **++** | **+** |  |
| **1071** | **-** | **-** | **-** | **-** | **-** | **-** | **-** |  |
| **1098** | **++** | **++** | **++** | **++** | **++** | **++** | **-** |  |
| **1118** | **+** | **+** | **+** | **-** | **++** | **++** | **++** |  |
| **1187** | **++** | **++** | **++** | **++** | **++** | **++** | **+** |  |
| **1299** | **++** | **++** | **++** | **++** | **++** | **++** | **+** |  |
| **1356** | **-** | **-** | **-** | **-** | **-** | **-** | **-** |  |
| **1357** | **-** | **-** | **-** | **-** | **-** | **-** | **-** |  |
| **1533** | **-** | **-** | **-** | **-** | **++** | **++** | **-** |  |
| **1534** | **++** | **++** | **++** | **++** | **++** | **++** | **+** |  |
| **1540** | **+** | **+** | **+** | **+** | **++** | **++** | **-** |  |
| **1541** | **-** | **-** | **-** | **-** | **-** | **-** | **-** |  |
| **1543** | **-** | **-** | **-** | **-** | **-** | **-** | **-** |  |
| **1544** | **-** | **-** | **-** | **-** | **-** | **-** | **-** |  |
| **1545** | **++** | **++** | **++** | **++** | **++** | **++** | **+** |  |
| **1547** | **++** | **++** | **++** | **++** | **++** | **++** | **+** |  |
| **1552** | **+** | **+** | **+** | **+** | **-** | **-** | **-** |  |
| **1553** | **+** | **+** | **+** | **+** | **++** | **++** | **+** |  |
| **1554** | **++** | **++** | **++** | **++** | **++** | **++** | **++** |  |
| **1557** | **++** | **++** | **++** | **++** | **++** | **++** | **+** |  |
| **1558** | **+** | **+** | **+** | **+** | **++** | **++** | **-** |  |
| **1559** | **++** | **++** | **++** | **++** | **++** | **++** | **+** |  |
| **1565** | **++** | **++** | **++** | **++** | **++** | **++** | **+** |  |
| **1566** | **++** | **++** | **++** | **++** | **++** | **++** | **+** |  |
| **1568** | **-** | **-** | **-** | **-** | **-** | **-** | **-** |  |
| **1571** | **-** | **-** | **-** | **-** | **-** | **-** | **-** |  |
| **157** | **++** | **++** | **++** | **++** | **+** | **+** | **-** |  |
| **1574** | **++** | **++** | **++** | **++** | **++** | **++** | **-** |  |
| **1575** | **++** | **+** | **+** | **+** | **++** | **++** | **+** |  |
| **1576** | **++** | **++** | **++** | **++** | **++** | **++** | **+** |  |
| **1578** | **++** | **++** | **++** | **++** | **++** | **++** | **+** |  |
| **1591** | **+** | **+** | **+** | **+** | **++** | **++** | **-** |  |
| **1626** | **-** | **-** | **-** | **-** | **-** | **-** | **-** |  |
| **1637** | **-** | **-** | **-** | **-** | **-** | **-** | **-** |  |
| **2000** | **++** | **+** | **+** | **+** | **+** | **+** | **-** |  |
| **2001** | **+** | **+** | **+** | **+** | **+** | **+** | **-** |  |
| **2033** | **++** | **++** | **++** | **-** | **++** | **++** | **+** |  |
| **2035** | **++** | **+** | **+** | **+** | **++** | **++** | **-** |  |
| **2114** | **+** | **+** | **-** | **-** | **-** | **-** | **-** |  |
| **2115** | **+** | **+** | **+** | **+** | **++** | **++** | **+** |  |
| **2118** | **-** | **-** | **-** | **-** | **-** | **-** | **-** |  |
| **2524** | **++** | **++** | **++** | **++** | **++** | **++** | **-** |  |
| **2111** | **++** | **++** | **+** | **++** | **++** | **++** | **+** |  |

"+", Peak area ≤10^4^; "++", Peak area>10^4^; "-" not detected; SRRC- Southern Regional Research Center

**Table S6. GenBank accession numbers for genomic sequences of 55 *Aspergillus* strains**

|  | **Genomic loci^a^** | | | |
| --- | --- | --- | --- | --- |
| **Strain** | ***amdS*** | ***benA*** | ***cmdA*** | ***trpC*** |
| 38 | MG825926 | MG825962 | MG826000 | MG826077 |
| 141 | MG825934 | MG825963 | MG826011 | MG826051 |
| 144 | MG825920 | MG825964 | MG826035 | MG826062 |
| 150 | MG825935 | MG825965 | MG826036 | N/A |
| 151 | MG825921 | MG825966 | MG826005 | MG826078 |
| 167 | MG825922 | MG825967 | MG826037 | MG826052 |
| 283 | MG825919 | MG825968 | MG826017 | MG826067 |
| 295 | MG825936 | MG825969 | MG826013 | MG826068 |
| 1000F | MG825937 | MG825970 | MG826038 | MG826079 |
| 1006 | MG825938 | MG825971 | MG826001 | MG826080 |
| 1020 | MG825927 | MG825972 | MG826002 | MG826081 |
| 1021 | MG825939 | MG825973 | MG826026 | MG826063 |
| 1055 | MG825923 | MG825974 | MG826039 | MG826053 |
| 1071 | MG825916 | MG825975 | MG826040 | MG826054 |
| 1098 | MG825940 | MG825976 | MG826027 | MG826082 |
| 1118 | MG825906 | MG825958 | MG825997 | MG826048 |
| 1187 | MG825929 | MG825977 | MG826028 | MG826083 |
| 1299 | MG825909 | MG825978 | MG825998 | MG826084 |
| 1356 | MG825930 | MG825979 | MG826029 | MG826085 |
| 1357 | MG825931 | MG825961 | MG826014 | MG826069 |
| 1533 | FJ877372 | MG825980 | MG826006 | FJ877157 |
| 1534 | FJ877442 | MG825981 | AY974341 | FJ877228 |
| 1540 | MG825924 | MG825982 | MG826041 | MG826086 |
| 1541 | MG825941 | JN394550 | MG826015 | MG826070 |
| 1543 | MG825913 | JN394552 | MG826018 | MG826087 |
| 1544 | MG825942 | MG825983 | MG826019 | MG826088 |
| 1545 | MG825928 | JN394554 | MG826003 | MG826089 |
| 1547 | MG825943 | JN394556 | MG826020 | MG826071 |
| 1552 | MG825944 | JN394561 | MG826016 | MG826072 |
| 1553 | MG825925 | JN394562 | MG826007 | MG826090 |
| 1554 | MG825945 | MG825984 | MG826042 | MG826055 |
| 1557 | MG825946 | MG825985 | MG826021 | MG826091 |
| 1558 | MG825947 | JN394567 | MG826030 | MG826056 |
| 1559 | MG825948 | JN394568 | MG826043 | MG826057 |
| 1565 | MG825908 | JN394574 | MG826031 | MG826058 |
| 1566 | MG825949 | JN394575 | MG825999 | MG826092 |
| 1568 | MG825910 | JN394577 | N/A | MG826073 |
| 1571 | MG825950 | JN394580 | MG826022 | MG826093 |
| 1573 | MG825912 | JN394582 | MG826004 | MG826059 |
| 1574 | MG825911 | MG825986 | N/A | MG826060 |
| 1575 | MG825951 | JN394584 | N/A | MG826074 |
| 1576 | MG825952 | MG825959 | MG826009 | MG826075 |
| 1578 | MG825915 | JN394586 | MG826010 | MG826094 |
| 1591 | MF521642 | MF521634 | MG826024 | MG826065 |
| 1626 | MG825932 | MG825987 | MG826012 | MG826049 |
| 1637 | MG825933 | MG825988 | MG826044 | MG826050 |
| 2000 | MG825953 | MG825960 | MG826032 | MG826095 |
| 2001 | MG825954 | MG825989 | MG826033 | MG826096 |
| 2033 | MG825907 | MG825990 | MG826025 | MG826066 |
| 2035 | MG825917 | MG825991 | MG826034 | MG826097 |
| 2111 | MG825914 | MG825992 | MG826023 | MG826076 |
| 2114 | MG825918 | MG825993 | MG826045 | MG826098 |
| 2115 | MG825955 | MG825994 | MG826046 | MG826061 |
| 2118 | MG825956 | MG825995 | MG826008 | MG826099 |
| 2524 | MG825957 | MG825996 | MG826047 | MG826064 |

^a^*amdS* (acetamidase), *benA* (beta-tubulin), *cmdA* (calmodulin), *trpC* (tryptophan synthase)

**Table S7. Species designations for 55 *Aspergillus* strains based on BLAST query of four unlinked genomic loci**

|  | **Genomic loci^a^** | | | |
| --- | --- | --- | --- | --- |
| **Strain** | ***amdS*** | ***benA*** | ***cmdA*** | ***trpC*** |
| 38 | *A. flavus* | *A. flavus* | *A. flavus* | *A. flavus* |
| 141 | *A. flavus* | *A. flavus* | *A. flavus* | *A. flavus* |
| 144 | *A. flavus* | *A. flavus* | *A. flavus* | *A. flavus* |
| 150 | *A. flavus* | *A. flavus* | *A. flavus* | *A. flavus* |
| 151 | *A. flavus* | *A. flavus* | *A. flavus* | *A. flavus* |
| 167 | *A. flavus* | *A. flavus* | *A. flavus* | *A. flavus* |
| 283 | *A. flavus* | *A. flavus* | *A. flavus* | *A. flavus* |
| 295 | *A. flavus* | *A. flavus* | *A. flavus* | *A. flavus* |
| 1000F | *A. flavus* | *A. flavus* | *A. flavus* | *A. flavus* |
| 1006 | *A. flavus* | *A. flavus* | *A. flavus* | *A. flavus* |
| 1020 | *A. flavus* | *A. flavus* | *A. flavus* | *A. flavus* |
| 1021 | *A. flavus* | *A. flavus* | *A. flavus* | *A. flavus* |
| 1055 | *A. flavus* | *A. flavus* | *A. flavus* | *A. flavus* |
| 1071 | *A. flavus* | *A. flavus* | *A. flavus* | *A. flavus* |
| 1098 | *A. flavus* | *A. flavus* | *A. flavus* | *A. flavus* |
| 1118 | *A. nomius* | *A. nomius* | *A. nomius* | *A. nomius* |
| 1187 | *A. flavus* | *A. flavus* | *A. flavus* | *A. flavus* |
| 1299 | *A. flavus* | *A. flavus* | *A. flavus* | *A. flavus* |
| 1356 | *A. flavus* | *A. flavus* | *A. flavus* | *A. flavus* |
| 1357 | *A. flavus* | *A. flavus* | *A. flavus* | *A. flavus* |
| 1533 | *A. flavus* | *A. flavus* | *A. flavus* | *A. flavus* |
| 1534 | *A. flavus* | *A. flavus* | *A. flavus* | *A. flavus* |
| 1540 | *A. flavus* | *A. flavus* | *A. flavus* | *A. flavus* |
| 1541 | *A. flavus* | *A. flavus* | *A. flavus* | *A. flavus* |
| 1543 | *A. flavus* | *A. flavus* | *A. flavus* | *A. flavus* |
| 1544 | *A. flavus* | *A. flavus* | *A. flavus* | *A. flavus* |
| 1545 | *A. flavus* | *A. flavus* | *A. flavus* | *A. flavus* |
| 1547 | *A. flavus* | *A. flavus* | *A. flavus* | *A. flavus* |
| 1552 | *A. flavus* | *A. flavus* | *A. flavus* | *A. flavus* |
| 1553 | *A. flavus* | *A. flavus* | *A. flavus* | *A. flavus* |
| 1554 | *A. flavus* | *A. flavus* | *A. flavus* | *A. flavus* |
| 1557 | *A. flavus* | *A. flavus* | *A. flavus* | *A. flavus* |
| 1558 | *A. flavus* | *A. flavus* | *A. flavus* | *A. flavus* |
| 1559 | *A. flavus* | *A. flavus* | *A. flavus* | *A. flavus* |
| 1565 | *A. flavus* | *A. flavus* | *A. flavus* | *A. flavus* |
| 1566 | *A. flavus* | *A. flavus* | *A. flavus* | *A. flavus* |
| 1568 | *A. flavus* | *A. flavus* | *A. flavus* | *A. flavus* |
| 1571 | *A. flavus* | *A. flavus* | *A. flavus* | *A. flavus* |
| 1573 | *A. flavus* | *A. flavus* | *A. flavus* | *A. flavus* |
| 1574 | *A. flavus* | *A. flavus* | *A. flavus* | *A. flavus* |
| 1575 | *A. flavus* | *A. flavus* | *A. flavus* | *A. flavus* |
| 1576 | *A. flavus* | *A. minisclerotigenes* | *A. flavus* | *A. flavus* |
| 1578 | *A. flavus* | *A. flavus* | *A. flavus* | *A. flavus* |
| 1591 | *A. flavus* | *A. parvisclerotigenus* | *A. flavus* | *A. flavus* |
| 1626 | *A. flavus* | *A. flavus* | *A. flavus* | *A. flavus* |
| 1637 | *A. flavus* | *A. flavus* | *A. flavus* | *A. flavus* |
| 2000 | *A. flavus* | *A. parvisclerotigenus* | *A. flavus* | *A. flavus* |
| 2001 | *A. flavus* | *A. flavus* | *A. flavus* | *A. flavus* |
| 2033 | *A. minisclerotigenes* | *A. flavus* | *A. flavus* | *A. flavus* |
| 2035 | *A. flavus* | *A. flavus* | *A. flavus* | *A. flavus* |
| 2111 | *A. flavus* | *A. flavus* | *A. flavus* | *A. flavus* |
| 2114 | *A. flavus* | *A. flavus* | *A. flavus* | *A. flavus* |
| 2115 | *A. flavus* | *A. flavus* | *A. flavus* | *A. flavus* |
| 2118 | *A. flavus* | *A. flavus* | *A. flavus* | *A. flavus* |
| 2524 | *A. flavus* | *A. flavus* | *A. flavus* | *A. flavus* |

^a^*amdS* (acetamidase), *benA* (beta-tubulin), *cmdA* (calmodulin), *trpC* (tryptophan synthase)
